# Novel Carbazole–Thiazole Conjugates: Synthesis and Biophysical Characterization

**DOI:** 10.3390/ijms26167945

**Published:** 2025-08-18

**Authors:** Beata Donarska, Klaudia Seklecka, Joanna Cytarska, Katarzyna Piechowska, Przemyslaw Ledwon, Sławomir Kula, Przemysław Krawczyk, Angelika Baranowska-Łączkowska, Krzysztof Z. Łączkowski

**Affiliations:** 1 Department of Chemical Technology and Pharmaceuticals, Faculty of Pharmacy, Collegium Medicum, Nicolaus Copernicus University, Jurasza 2, 85-089 Bydgoszcz, Poland; beata.donarska@cm.umk.pl (B.D.); cytar@cm.umk.pl (J.C.); kpiechowska@cm.umk.pl (K.P.); 2 Department of Physical Chemistry and Technology of Polymers, Faculty of Chemistry, Silesian University of Technology, Strzody 9, 44-100 Gliwice, Poland; przemyslaw.ledwon@polsl.pl; 3 Institute of Chemistry, Faculty of Science and Technology, University of Silesia, Szkolna 9 St., 40-007 Katowice, Poland; slawomir.kula@us.edu.pl; 4 Department of Physical Chemistry, Faculty of Pharmacy, Collegium Medicum, Nicolaus Copernicus University, Kurpińskiego 5, 85-950 Bydgoszcz, Poland; przemekk@cm.umk.pl; 5 Faculty of Physics, Kazimierz Wielki University, Powstańców Wielkopolskich 2, 85-090 Bydgoszcz, Poland

**Keywords:** carbazole, thiazole, tyrosinase, photoluminescence, cyclic voltammetry, TD-DFT method, optical materials, thermogravimetric analysis

## Abstract

This presented study depicts the synthesis of three novel carbazole–thiazole conjugates, thoroughly investigating their spectroscopic properties as well as evaluating their biological activity as tyrosinase inhibitors. Additionally, we investigated the possibility of using Concanavalin A (ConA) complexes with dyes from a theoretical point of view, developing a promising protein-based strategy of delivery of dyes to the target cells. The tyrosinase inhibition assay showed that compounds **K1** and **K3** demonstrated higher activity than the kojic acid with IC_50_ values of 46 and 59 mM, respectively. Among the tested compounds, carbazole **K3** exhibits the most pronounced nonlinear optical response due to its high electronic flexibility, strong solvatochromism, large excited-state dipole moments, and efficient intramolecular charge transfer. Additionally, all investigated carbazoles demonstrate high ability to form stable supramolecular complexes with ConA, which was confirmed using molecular docking studies. It was found experimentally and theoretically that the compound **K3** has the best biophysical parameters, making it a promising candidate for potential diagnostic applications.

## 1. Introduction

Carbazoles are heterocyclic aromatic compounds containing a tricyclic system consisting of two benzene rings joined by a five-membered ring containing nitrogen atom. Due to their unique chemical structure, carbazoles exhibit interesting physicochemical properties, which make them the subject of intensive research in various scientific fields—from materials chemistry through pharmacology to bioengineering. These compounds are attracting special attention from scientists due to their high thermal stability, strong fluorescence properties, and ability to be easily chemically modified, which enables tailoring their properties to specific applications [1].

In medicine, carbazoles exhibit a wide range of biological activity, including anti-inflammatory, antibacterial, antifungal, antiviral, and antioxidant effects [2,3]. There is a great interest in the anticancer properties of carbazoles, which are associated with various mechanisms of action [4,5]. A classic example of a natural compound from this group is ellipticine, an alkaloid with the ability to inhibit topoisomerase II and DNA intercalation [6]. Some carbazole derivatives have been identified as potential inhibitors of protein kinases—enzymes that play a key role in cancer pathogenesis. Recently, murrayanine, another carbazole alkaloid, has been shown to inhibit the growth of oral cancer cells by modulating the AKT/mTOR and Raf/MEK/ERK signaling pathways, thereby limiting tumor growth in vivo [7]. Synthetic carbazoles, such as derivatives of thiazolidinone, indoles, and carbaldehyde, have been shown to inhibit the growth of liver, breast, colon, and melanoma cancer cells. In the context of therapy of the latter type of cancer, of particular interest is the ability of carbazoles to inhibit activity of tyrosinase—an enzyme that catalyzes hydroxylation of L-tyrosine to L-DOPA and its further oxidation to L-DOPA-quinone.

Excessive activity of this enzyme is associated with pigmentation disorders (such as melasma, age spots), as well as with the pathogenesis of melanoma and chronic skin inflammation. Recently, 2-benzimidazole, benzothiazole, and benzoxazole carbazole derivatives have been described as potent competitive tyrosinase inhibitors that bind to the active site of tyrosinase via interaction with its dinuclear copper center [8]. In melanoma therapy, the ability of compounds to inhibit tyrosinase activity is associated with the sensitivity of cancer cells to radiotherapy and chemotherapy, by reducing the production of cancer cells protecting melanin. In addition to their use in cancer therapy, the ability to inhibit tyrosinase is also associated with the possibility of using carbazole derivatives in the food and materials industry [9].

The applications of carbazoles in molecular biology and biochemistry as building blocks for fluorescent sensors are equally interesting. Due to their optical properties, carbazoles can be used as probes in studies on enzymes, protein structures, and intermolecular interactions. The carbazole ring is characterized by chemical and photostability, as well as the possibility of introducing various functional groups, which allows for the appropriate adjustment of fluorescence emission both for the detection of metal ions, changes in environmental pH, and the detection of specific enzymatic reactions. Fluorescent probes based on the carbazole structure are characterized by high selectivity towards biological targets and detection sensitivity, which allows detection of low concentrations of analytes [10]. Recently, a fluorescent probe based on a carbazole structure has been presented for the detection of fluoride ions in biological samples, which is important due to the toxic effects of excessive fluoride concentrations in the human body [11]. Another example is the fluorescent probe designed by Wang et al., based on a carbazole structure conjugated with thiophene, capable of selective detection of copper II ions in aqueous solutions and in living cells. The probe has been shown to easily penetrate biological membranes and exhibit low toxicity, making it a useful tool in biomedical research [12].

The next, equally important application of carbazole compounds is their use in organic electronics, primarily as light-emitting materials in organic light-emitting diodes (OLEDs), as well as components of active layers in organic photovoltaic cells (OPVs) and thin-film transistors (OTFTs). The carbazole structure is characterized by favorable properties such as electron delocalization in the conjugated π system and good hole transport, high thermal and electrochemical stability, high photoluminescence quantum yield (PLQY), and ease of functionalization in various places, which makes it an attractive material in terms of applications in optics and electronics [13]. Recently, new derivatives of carbazole conjugated with benzo[a]carbazole have been published, exhibiting excellent thermal and optical stability, serving as dedicated host materials in OLEDs, as well as improving the efficiency and lifetime of diodes [14].

Considering the above-mentioned findings, we decided to continue the search for new compounds with more favorable properties as dyes and with larger biological activity. We decided to achieve this effect by introducing into the molecule the cyano (-CN) and nitro (-NO_2_) groups, which are commonly used in dyes as electron-withdrawing groups. Additionally, we also decided to investigate the effect of the large, rigid electron-donating adamantyl group, which has the ability to bind to the hydrophobic pocket of enzymes. Our research includes detailed investigation of the substituent effect on spectroscopic properties, and thus the possibility of using these materials as sensitive molecular probes and potential anticancer drugs based on the mechanism of tyrosinase inhibition. Additionally, we investigated the possibility of using Concanavalin A (ConA) complexes with dyes, developing a promising protein-based strategy of delivery of dyes to the target cells, as well as a way to improve probe efficiency.

## 2. Results and Discussion

### 2.1. Chemical Synthesis

**Scheme 1:**
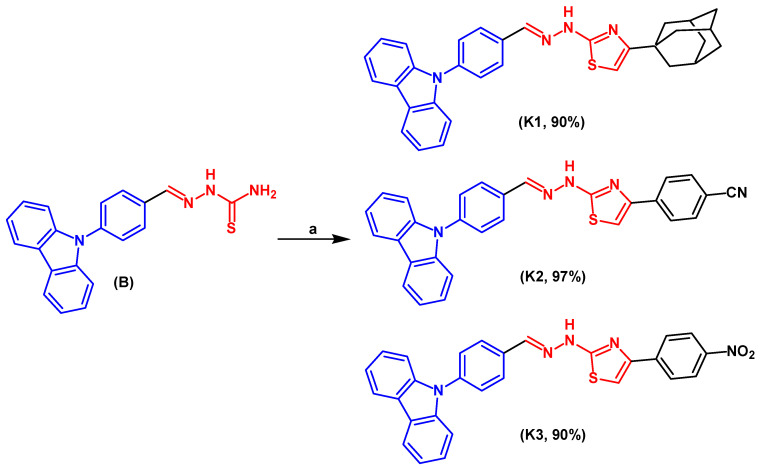
General synthesis of compounds **K1**–**K3**: Reagents and conditions: (a) 2-bromoketones, EtOH, reflux, 20 h.

The synthesis of new carbazole derivatives containing thiazole moiety **K1**–**K3** was carried out using the reaction of 2-(4-(9H-carbazol-9-yl)benzylidene)hydrazinecarbothioamide (**B**), prepared according to the known procedure [15], and commercially available 2-bromoketones in ethanol solvent for 20 h under reflux. The targeted products **K1**–**K3** were obtained with 90–97% yield, and their synthesis is outlined in Scheme 1. All products were purified on silica gel column chromatography and fully characterized employing spectroscopic methods, ^1^H, ^13^C NMR, and ESI-HRMS analysis (see Supplementary Information). The ^1^H NMR peaks at 6.45, 7.67, and 7.76 ppm are characteristic signals from the thiazole ring-5H protons, confirming obtaining of target products. Additionally, bold signals from the NH group can be found in the range of 12.34–12.43 ppm. All products also have their characteristic peaks corresponding to their molecular [M+H]^+^ ions, observed in the ESI-HRMS spectra.

### 2.2. Mushroom Tyrosinase Inhibition

Carbazoles **K1**–**K3** were examined for their inhibitory effects on mushroom tyrosinase using L-DOPA as substrate. The results are summarized in Table 1. The results were compared with standard tyrosinase inhibitors, namely kojic acid and ascorbic acid.

All tested compounds showed higher effect of tyrosinase inhibition than ascorbic acid (IC_50_ 386.50 µM). Among the investigated carbazoles, the best inhibitory effect was demonstrated by compound **K3**. Its IC_50_ value of 45.95 µM means 1.6 times higher inhibitory effect than kojic acid (IC_50_ 72.27 µM) and more than 8 times higher inhibitory effect than ascorbic acid (IC_50_ 386.5 mM). This compound is characterized by the presence of the 4-nitrophenyl group. Almost comparable activity to **K3** was shown by the **K1** compound containing the adamantyl moiety, with IC_50_ value equal to 59.36 µM. Slightly lower tyrosinase inhibition effect than kojic acid was shown by compound **K2** (IC_50_ 81.99 µM), containing the cyano group.

#### Kinetic Analysis of the Inhibition of Tyrosinase

**Figure 1:**
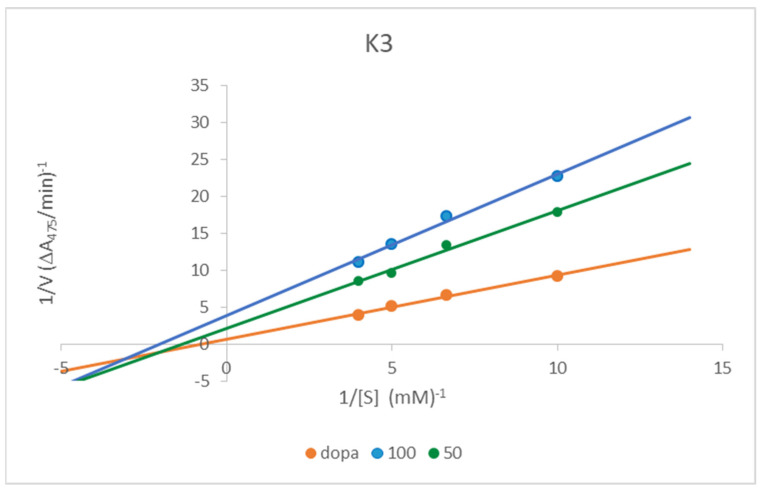
Lineweaver–Burk plot for inhibition of tyrosinase for tested **K3**. The plot is expressed as 1/velocity [ΔA_475_/min^−1^] vs. 1/substrate concentration [mM^−1^].

Based on the Lineweaver–Burk double reciprocal plots, the inhibition mode for the three investigated compounds was established as mixed-type inhibition, i.e., the type of enzyme inhibition in which the inhibitor may bind to the enzyme regardless of whether or not the enzyme has already bound the substrate. Out of all the investigated compounds, the **K2** carbazole is characterized by the lowest value of the inhibition constant, K_M_ 0.314, which corresponds to the high enzyme affinity for substrate (Figure 1).

### 2.3. Physicochemical Properties

#### 2.3.1. Thermal Stability

**Figure 2:**
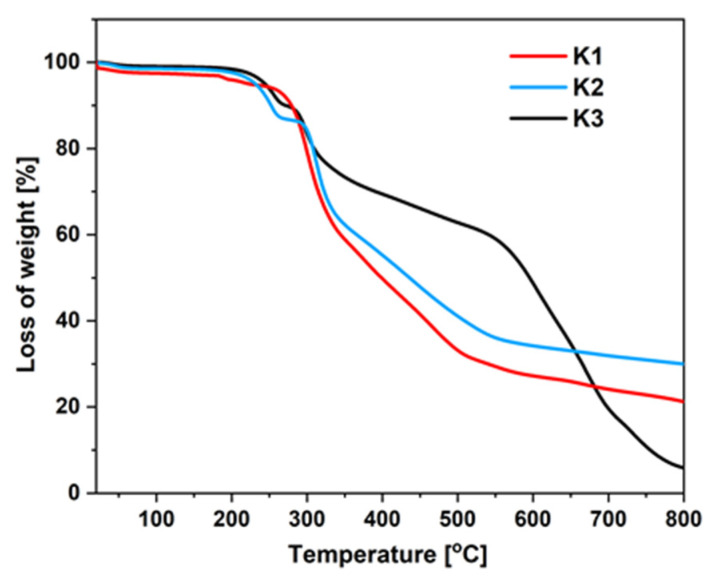
Thermogravimetric analysis thermogram of **K1**–**K3**.

Thermogravimetric analysis (TGA) allowed obtaining of thermal stability results for the presented compounds (**K1**–**K3**). For all derivatives, the temperature of 5% (T5) and 10% (T10) mass loss as well as the temperature of complete decomposition (T_max_) were determined. The collected data are presented in Table 2 and Figure 2. Referring to the T5 for **K1**–**K3**, we can see that it is in the range of 222 °C to 244 °C. Such high values indicate the high durability of these compounds. Moreover, the functional group in the substituent has a negligible effect on thermal stability. The best thermal properties are those of **K3** compound, containing the 4-nitrophenyl group. In the case of replacing this group with benzonitrile (**K2**), we observe a slight decrease in T5 (by 13 °C), T10 (by 19 °C), and only one stage of Tmax degradation. On the other hand, compound **K1** has the lowest T5 (222 °C). It is most likely due to the aliphatic nature of the substituent. Comparing the discussed derivatives (**K1**–**K3**) to their analogs described in the literature, we can see a significant similarity in thermal properties, despite different functional groups in the substituents [15].

#### 2.3.2. Photoluminescence Properties

**Figure 3:**
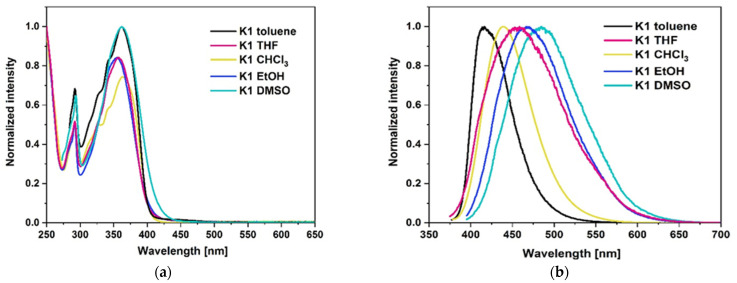
Absorption (**a**) and emission (**b**) spectra for **K1**.

Absorption and emission studies were carried out for investigated compounds **K1**–**K3** using solvents of different polarities. The presented molecules differ in the substituent in thiazole ring. To check the influence of the substituent in the discussed molecules, the tests were carried out on solutions with a concentration of 2.5 × 10^−5^ mol/L. In the first step, absorption maxima (λ_abs_) were determined. The measured data are shown in Table 3, Figure 3, Figures S1 and S2.

Each of the derivatives has at least two bands registered in the absorption spectra. The main bands between 320 and 370 nm can be attributed to the π→π* transition. The next one is the band above 300 nm. In the case of the presented molecules, the solvatochromic effect associated with the polarity of the solvent is not observed in the absorption spectra. The same behavior is shown by the analogs described in the literature [15,16]. In the next step, the emission maxima were determined. For the **K3** molecule, spectra were recorded only in chloroform, ethanol, and DMSO. This compound did not dissolve in non-polar toluene and did not give an emission spectrum in THF. For the rest, λ_em_ was successfully determined in several solvents. By analyzing the fluorescence spectra, the influence of the polarity of the solvent on the obtained results can be observed. In compounds **K1**–**K3**, a red shift was observed with increasing solvent polarity. Moreover, the Stokes shift increases from non-polar to polar solvents (**K3** 30 nm, **K1** 69 nm, **K2** 70 nm). This is a typical effect for compounds with a different charge distribution in the excited state compared to the ground state. This suggests greater stabilization of the excited singlet state in the polar environment. Such behavior shows compounds with CT (charge transfer) character for which excitation causes an increase in the dipole moment [16,17,18]. The presented molecules are very similar to their analogs described in the literature [15,16]. Comparing the results obtained in chloroform, **K3** and **K1** compounds are the most blue-shifted relative to their structural analogs.

#### 2.3.3. Cyclic Voltammetry Measurements

**Figure 4:**
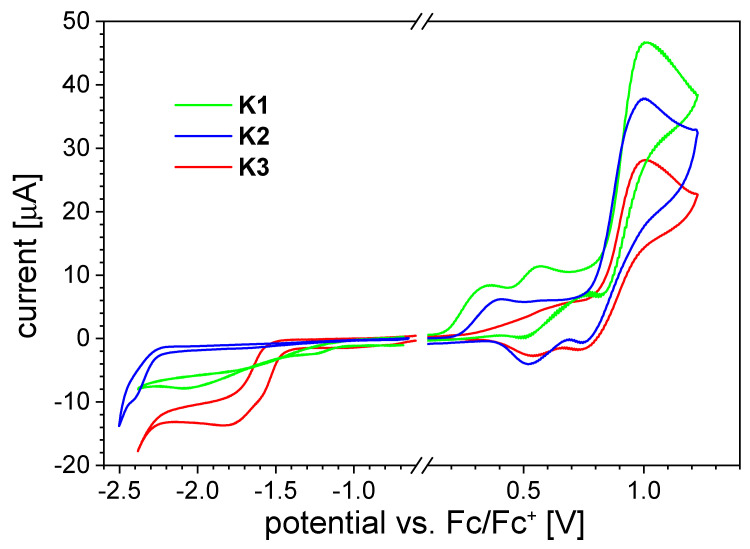
Cyclic voltammetry of **K1**, **K2**, and **K3** compounds in Bu_4_NBF_4_/CH_2_Cl_2_ electrolyte.

The influence of the chemical structure on electrochemical properties was estimated using cyclic voltammetry characterization. This technique is considered as a basic tool for the characterization of conjugated organic molecules, including the estimation of possible coupling processes that can lead to formation of conjugated polymers [19]. The cyclic voltammetry of the studied compounds is shown in Figure 4. The reduction and oxidation curves exhibit complex behavior, which can be attributed to the intricate structure of the studied compounds. The compounds contain carbazole as the main electroactive unit, with electroactive hydrazine-based side chains linked to carbazole via a nitrogen atom. This structure allows for the oxidation and reduction of both the carbazole and the hydrazine-based group.

**Figure 5:**
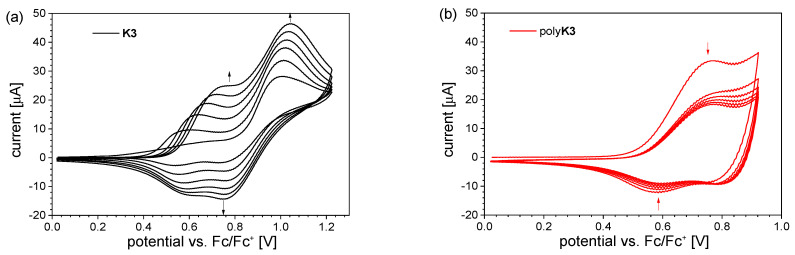
Cyclic voltammetry of (**a**) 1 mM solution of **K3** in Bu_4_NBF_4_/CH_2_Cl_2_ electrolyte and (**b**) electrochemically generated poly (**K3**) film in monomer free electrolyte.

The oxidation of all compounds starts at a similar potential. When comparing the oxidation onset potentials of the tested compounds, it can be concluded that the differences are not significant. The onset of oxidation in compound **K1** is 0.18 V, for compound **K2**, it is 0.22 V, and the highest oxidation potential of 0.28 V is observed for compound **K3**. Additionally, the shape of the observed voltammograms in the anodic range is similar for each of the tested compounds. First, a double wave is observed, indicating a two-stage oxidation. Then, a peak with a higher current at 1 V for all compounds is observed. Its potential is similar to the oxidation potential of the analogous N-phenylcarbazoles [20]. This clearly indicates that this peak is related to the oxidation of the N-substituted carbazole unit, while the earlier oxidation waves are related to the reactions on the hydrazine- and thiazole-based moieties [15]. After changing the direction of the potential sweep, reduction peaks are also observed. They are asymmetric in relation to the oxidation peaks, which indicates the electrochemical irreversible nature of the oxidation process. These waves indicate the coupling of two carbazole units, which is characteristic of this group of chemical compounds [21]. It was decided to confirm this process by registering multiple voltammetric cycles. As shown in Figure 5 the carbazole coupling process of **K3** is visible as a rising peak at 0.6–0.75 V potential.

A similar process is also observed for N-phenylcarbazole derivatives [20]. When examining the reduction processes, the complex nature of the voltammetry curves in the cathodic range is also observed. Significant differences in the onset of reduction for the tested compounds are observed. Reduction starts at −1.13 V for **K1**, −1.48 V for compound **K3**, and −2.27 V for compound **K2**. Moving towards lower potentials, successive waves of reduction are observed. This behavior can be attributed to the occurrence of azo-hydrazone tautomerism of the C=N-NH hydrazone group [22,23].

### 2.4. Computational Evaluation of Molecular Behavior

#### 2.4.1. Molecular and Spectroscopic Characteristics

Natural Bond Orbital (NBO) analysis (Table S1), which quantifies electron distribution in chemical bonds, reveals that the substituent attached at position C38 (Figure S3) critically modulates the stability of the carbazole-based complexes (molecular backbone: (*E*)-2-(2-(4-(9*H*-carbazol-9-yl)benzilidene)hydrazinyl)thiazole). The **K3** complex exhibits the lowest LUMO energy (−2.89 eV in water, Table S2), while **K1** shows the highest (−5.70 eV in toluene), directly correlating with their electronic stabilization trends. Orbital energy analysis demonstrates that **K3** substituent, -NO_2_, provides the strongest stabilization (LUMO energy: −2.89 eV in water), while **K1** is the least stable (LUMO: −5.70 eV in toluene). This 2.81 eV difference reflects **K3** efficient electron delocalization. Electron occupancy in each complex remains at a similar level, 1.97 eV, but subtle differences may result from the inductive effect of the substituents. The charge on the C38 atom decreases in the order **K1** > **K3** > **K2**, which may indicate differences in the electron-donating nature of the substituents. The solvent can influence the stability of the system through electrostatic interactions and solvation effects. Solvent polarity is responsible for stabilization: in water, orbital energy of the **K3** decreases by 0.52 eV compared to toluene, reflecting enhanced charge delocalization in polar media. This effect is the weakest for **K1** (ΔE = 0.12 eV), indicating its low solvation sensitivity. The charge on the C38 atom tends to decrease in more polar solvents, indicating electron redistribution under solvation influence. Bond polarization changes minimally in highly polar solvents, suggesting a more stable bond character in such environments. For the **K1**, orbital energy changes the least depending on the solvent, indicating its greater resistance to solvation effects compared to **K2** and **K3**. The substituent in **K3** stabilizes the structure the most, which is evident in lower orbital energies, while solvents with higher polarity, such as H_2_O and DMSO, stabilize the molecules by lowering orbital energy and redistributing the charge on C38. Bond polarization shows slight variations across different solvents, suggesting that the electronic properties of the complexes are relatively stable. The **K2** and **K3** complexes are more susceptible to solvent influence than K1, indicating greater flexibility of their electronic systems.

The graphical analysis of HOMO and LUMO orbitals for the tested compounds (Figure S4) reveals significant differences in electron density distribution influenced by the substituents. For **K1**, the HOMO orbital is concentrated on the carbazole part, indicating that the donor electron density is focused in the center of the molecule, while the LUMO is more delocalized, potentially facilitating interactions with electron acceptors. In the case of **K2**, a shift of the HOMO towards the substituent is observed, suggesting its active role in electron delocalization, while the LUMO is more localized in peripheral regions, which may affect its reactivity. For **K3**, the LUMO orbital is the most extended, and the HOMO shows a clearly decentralized electron density, suggesting greater electron mobility and increased chemical reactivity compared to **K1** and **K2**. These changes confirm previous energy analysis results, indicating greater stability for **K1** and higher reactivity and electronic flexibility for **K3**.

Based on the analysis of HOMO–LUMO energy levels and related chemical parameters for the **K1**, **K2**, and **K3** compounds in various solvents (Table S2), a clear influence of both substituents and solvent environment can be observed. **K3** displays the lowest LUMO and highest HOMO energies, resulting in the narrowest energy gap (ΔE_GAP_), which indicates higher reactivity and lower kinetic stability. In contrast, **K1** has the widest gap, suggesting greater stability and reduced reactivity. Increasing solvent polarity slightly reduces ΔE_GAP_, indicating enhanced electron delocalization and stabilization in polar environments. This effect is most notable for **K3**, where ΔE_GAP_ drops from toluene to water, implying improved conditions for electronic transitions. Chemical hardness (η) and softness (σ) confirm these observations. **K1** is the hardest and least reactive, while **K3** is the softest and most reactive. **K3** also shows the highest electronegativity (χ), chemical potential (μ), and global electrophilicity (ω), making it more prone to electrophilic interactions. **K1**, with its higher hardness and ΔE_GAP_, has a lower tendency to participate in these reactions. The influence of solvents is also evident in these parameters, especially for **K3**, where polar solvents tend to lower orbital energies and enhance electrophilicity. **K2** is still characterized by intermediate values, suggesting a balance between reactivity and stability in all solvents. In summary, **K3** is the most reactive due to its narrow energy gap and high electrophilicity, while **K1** is the most stable and least sensitive to solvent effects. Therefore, polar environments significantly increase stabilization and can enhance the electronic properties of the studied molecules used in chemical or optoelectronic systems.

Based on the Molecular Electrostatic Potential (MEP) surfaces (Figure S5) for the **K1**, areas with the most negative potential, indicating increased electron density, are primarily concentrated around the carbazole part, confirming previous conclusions about its stability and lower reactivity. **K2** exhibits a more varied distribution of electrostatic charge, with a greater influence of substituents in modulating electronic properties, which aligns with the observed greater delocalization of the HOMO in this molecule. **K3** is characterized by the broadest spread of regions with high electron density, consistent with previous findings regarding its greater electronic flexibility and susceptibility to electrophilic reactions. Therefore, the higher stability of **K1** and the increased chemical reactivity and greater electron delocalization of **K3** are confirmed.

The changes in electron density upon photoexcitation (Δρ (r)) are graphically shown in Figure S6. In the case of **K1**, regions of electron density depletion (blue) are concentrated around the carbazole part, while the zones of electron density increase (purple) are distributed mainly over the π–electron bridge, completely omitting the adamantate substituent. This indicates limited charge mobility within the molecule and aligns with its previously described stability. In **K2**, a more pronounced asymmetry between the depletion and accumulation regions is observed, pointing to greater charge polarization and more dynamic electron redistribution influenced by the substituents. **K3** exhibits the broadest distribution of electron depletion and accumulation zones, suggesting increased charge mobility and a greater tendency for electron transfer. The analysis of the amount of transferred charge (q_CT_) and charge transfer distance (D_CT_, Table S3) confirms these observations and reveals the influence of both substituents and solvents. q_CT_ values increase in the order **K1** < **K2** < **K3**, indicating a progressive rise in the amount of transferred charge, which is in accordance with the decreasing power of deactivating substituents. **K1** shows the lowest q_CT_ values, suggesting greater electronic stability and less tendency toward charge transfer, while **K3** exhibits the highest values, reflecting more dynamic electronic behavior and enhanced donor–acceptor interactions. Similarly, D_CT_ values follow the same trend, with **K3** displaying the longest transfer distances, supporting its more delocalized electronic nature. The solvent effect is particularly evident in D_CT_ values. For **K1**, D_CT_ decreases with increasing solvent polarity, implying a more compact charge distribution and limited electron reorganization in polar environments. In contrast, for **K2** and **K3**, increasing polarity results in greater D_CT_ values, which reflects increased electronic flexibility and facilitated charge displacement. For **K3**, the differences are less pronounced, suggesting that this molecule is already inherently more delocalized and less sensitive to environmental changes compared to **K1** and **K2**.

The analysis of free energy of solvation (ΔG_solv_, Table S4) for the tested compounds indicates a clear influence of both molecular structure and solvent type on the stabilization of these systems in solution. For all three molecules, the most negative ΔGsolv values are observed in toluene and chloroform, indicating better stabilization in non-polar and moderately polar organic environments. As the polarity of the solvent increases, the solvation energy becomes less negative, reflecting weaker stabilization of the compounds. **K1** shows the greatest variation in solvation energy depending on the solvent, from −27.45 kcal/mol in TCM to −10.88 kcal/mol in H_2_O. This suggests that the structure of this compound is most sensitive to changes in the surrounding environment. **K2** exhibits the lowest solvation energy in TCM (−29.67 kcal/mol), indicating a particularly favorable interaction with this solvent. In contrast, **K3** generally displays less negative ΔG_solv_ compared to **K1** and **K2**, suggesting that it is the least stabilized by solvation and has greater freedom in charge redistribution within the molecule.

The calculated vertical absorption maxima (λ_abs_, Table S5) for the **K1**, **K2**, and **K3** molecules show good agreement with experimental values. In particular, very comparable results were obtained for **K1**, with an average deviation of only 2.2 nm. For **K3**, the error is moderate (4.8 nm), while the largest discrepancies are observed for **K2** (6.6 nm). Compared to cLR approximation, vertical values yields consistently lower mean errors across all three derivatives, with the exception of **K3**, where both approaches perform similarly. However, vertical values shows greater consistency overall and more accurately reproduces spectral trends, confirming its reliability for describing the optical properties of the studied molecules. The absorption maxima also indicate the presence of solvatochromism. **K1** and **K3** exhibit positive solvatochromism, with absorption maxima shifting to longer wavelengths as solvent polarity increases; for **K1**: from 357 nm in THF to 362 nm in DMSO; for **K3**: from 356 nm in EtOH to 362 nm in DMSO. In contrast, **K2** shows negative solvatochromism, with a clear hypsochromic shift in more polar solvents, from 367 nm in TCM to 342 nm in EtOH. These shifts correlate well with the calculated dipole moments of the ground (μ_GS_) and excited (μ_CT_) states (Table S6). **K3** shows the largest increase in dipole moment, ranging from 7.66 D in toluene to 9.16 D in water for the ground state and from 35.21 D to 37.02 D for the excited state, depending on the solvent. This is followed by **K2**, whose dipole moment ranges from 6.38 D to 7.42 D in the ground state and from 17.94 D to 18.92 D in the excited state. The smallest values are observed for **K1**, with dipole moments ranging from 4.14 D to 4.26 D in the ground state and from 8.26 D to 8.34 D in the excited state. An increase in the polarity of the excited state (〖Δμ〗_(CT-GS)) reflects a redistribution of charge upon excitation, which is characteristic of charge transfer (CT) transitions. The greater the difference in these values, the stronger the CT character, and the higher the sensitivity of the system to solvent polarity. In the case of **K1** and **K3**, which exhibit positive solvatochromism, the high dipole moments in the excited state indicate that this state is more stabilized by polar solvents. On the other hand, for **K2**, where a blue shift is observed, the smaller dipole moment difference suggests that the ground state is more stabilized in polar environments. Thus, the dipole moment values reflect the direction of the absorption band shift and confirm that for **K3**, the largest increase in dipole moment corresponds to the most pronounced polarity-dependent spectral shift. This confirms the strong charge transfer (CT) character of the excited state for **K3**, in agreement with the density difference maps and the highest q_CT_ and D_CT_ values, indicating substantial electronic redistribution upon excitation.

**Figure 6:**
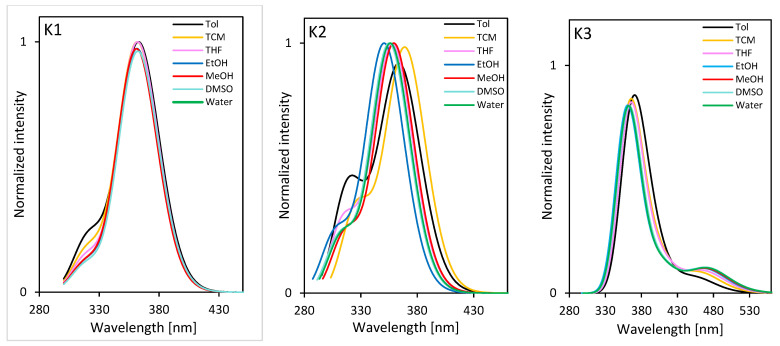
Theoretical UV–Vis absorption spectra of **K1**–**K3** obtained from TDDFT/PBE0 calculations, illustrating solvent effects on electronic transitions.

Comparison of the graphically presented absorption spectra obtained experimentally and theoretically (Figure 6) reveals a good match in the position of the main absorption bands across all three derivatives. Theoretical spectra also predict additional low-intensity absorption bands, particularly in the 310–340 nm region. These bands likely originate from n→π* or π→π* transitions localized on substituents. Their presence is supported by the significant changes in dipole moments and the polarized nature of the CT transitions. This is especially true for **K3**, where the 〖Δμ〗_(CT-GS) exceeds 30 D, suggesting that these weak transitions may meaningfully contribute to the overall spectral shape.

The theoretical emission maxima (λ_em_, Table S7) also correlate well with experimental data. **K1** and **K3** show small differences, while for **K2**, the calculated maxima are slightly higher than measured. Increasing solvent polarity results in a red shift of λ_em_, consistent with the behavior of CT excited states. **K1** emits in the 416–490 nm range, **K2** in 432–471 nm, and **K3** in 444–477 nm. Theoretical values predict slightly larger shifts but follow the same directional trend. Stokes shifts values are largest for **K1** (up to 7300 cm^−1^ in water) and **K3** (~6600 cm^−1^), indicating substantial structural relaxation in the excited state. These large shifts reflect strong excited-state stabilization and are consistent with the significant dipole moment increases and CT characteristics observed in these systems.

The molecule **K3** exhibits the highest first hyperpolarizability values (β, Table S8) among all the studied systems, ranging from 3503.14 a.u. in toluene to 8796.30 a.u. in water, significantly exceeding those of **K1** (from 890.71 to 1877.40 a.u.) and **K2** (from 1000.44 to 2974.45 a.u.). These differences are consistent across all solvents, and as solvent polarity increases, a pronounced enhancement in β is observed, particularly for **K3**, where the increase exceeds 5200 a.u. Similarly, **K3** also shows the highest isotropic polarizability (α), with values ranging from 282.60 a.u. in toluene to 307.91 a.u. in water, confirming its superior electronic responsiveness. The polarizability values for **K1** (239.77–248.69 a.u.) and **K2** (254.28–264.20 a.u.) remain lower throughout, reflecting more rigid and less polarizable electron distributions.

Carbazole **K3** exhibits the most pronounced nonlinear optical response among the studied compounds, attributed to its high electronic flexibility, strong solvatochromism, large excited-state dipole moments, and efficient intramolecular charge transfer, as confirmed by density difference maps and q_CT_/D_CT_ values. Its batochromic-shifted spectra, large Stokes shifts, and polarized electron distribution further support CT character. In contrast, **K1** and **K2** show lower polarizability, smaller dipole changes, and limited NLO potential. Considering both β values and spectroscopic consistency, **K3** emerges as the most promising candidate for applications such as SHG, nonlinear imaging probes, and bioimaging.

#### 2.4.2. Delivery of Compounds

Fluorescent probe-based imaging techniques of biological systems are currently recognized as one of the most powerful methods used in medicinal chemistry. Proteins occupy a special place due to their natural biocompatibility, increased solubility, and bioavailability of fluorescent probes [24]. Concanavalin A (ConA) belongs to the lectin family proteins designated by specificity for strong binding polysaccharides containing α-glucose, mannose, glucosamine, and α-N-acetylglucosamine. These glycans are present on the surface of all types of cells and play roles in the immune system and cellular signaling. ConA, originally extracted from the jack-bean (Canavalia ensiformis), is a homotetramer at neutral pH [25]. Recently it was reported that the ConA complex with Rose Bengal photosensitizer has been successfully used in targeted photodynamic therapy against Gram-negative bacteria [26]. For this reason, we decided to investigate the possibility of creating supramolecular complexes of ConA and Human serum albumin (HAS) with compounds **K1**–**K3** in order to direct the photoactive compound to saccharides on the cell wall to increase their potency.

It was found that all investigated carbazole derivatives demonstrate the ability to form stable supramolecular complexes with model proteins, including ConA and HSA, involving lysine residues (LYS) as binding centers. In the interaction of the studied carbazole derivatives with ConA (Table S9), molecular docking analysis reveals differences in both the binding site location and the binding free energy values. **K1** binds within the region centered around LYS116, while for **K2** and **K3**, the binding occurs at LYS46. The most favorable binding free energy (ΔG_b_) is observed for **K1** and amounts to −6.30 kcal/mol, indicating the highest affinity toward ConA. For **K2** and **K3**, the ΔG_b_ values are slightly higher, at −5.40 kcal/mol and −5.30 kcal/mol, respectively. This suggests slightly weaker, but still beneficial, interactions with the active site. The change in binding site from LYS116 (for **K1**) to LYS46 (for **K2** and **K3**) may result from steric and electronic differences caused by the substituents at the C38 position. In each case, the molecule is not stabilized by hydrogen bonds or π–π* interactions, indicating that the main forces responsible for binding to the macromolecule are electrostatic interactions and geometric complementarity of the probe to the aromatic cavity of the protein. In the case of HSA (Table S10), all three compounds bind to the same active site (LYS444), with **K1** again exhibiting the strongest binding (ΔG_b_ = −10.3 kcal/mol), and **K2** and **K3** showing comparable values (−9.0 kcal/mol each). In all cases, conjugation occurs without additional stabilizing interactions such as hydrogen bonding or π–π* stacking.

## 3. Materials and Methods

All details regarding UV-Vis absorption measurements [27], and the computational procedure [28,29,30,31,32,33,34,35,36,37,38,39,40,41,42,43,44,45,46,47,48,49,50,51,52], have been placed in the Supplementary Materials.

### 3.1. Measurement

All experiments were carried out under air atmosphere unless stated otherwise. Reagents were generally the best quality commercial-grade products and were used without further purification ^1^H NMR (400 MHz) and ^13^C NMR (100 MHz) spectra were recorded on a Bruker Avance III (Billerica, MA, USA) multinuclear instrument. High-resolution mass spectrometry measurements were performed using Synapt G2-Si mass spectrometer (Waters, Milford, MA, USA) equipped with quadrupole Time-of-flight mass analyzer. The mass spectrometer was operated in the positive ion detection mode. The results of the measurements were processed using the MassLynx 4.1 software (Waters, Milford, MA, USA) incorporated with the instrument. Melting points were determined in open glass capillaries and are uncorrected. Analytical TLC was performed using Macherey-Nagel (Düren, Germany) Polygram Sil G/UV254 0.2 mm plates.

#### General Experimental Procedure for Synthesis of Carbazole **K1**–**K3**

The synthesis of the new compounds **K1**–**K3** was carried out according to the procedure described previously [15].

(*E*)-2-(2-(4-(9*H*-carbazol-9-yl)benzylidene)hydrazinyl)-4-(1-adamantyl)thiazole (**K1**). Yield: 0.45 g, 90%, (dichloromethane/methanol (95:5), R_f_ = 0.86); mp 198–204 °C. ^1^H NMR (700 MHz, DMSO-d_6_), δ (ppm): 1.67–1.78 (m, 6H, 3CH_2_); 1.86–1.93 (m, 6H, 3CH_2_); 2.01–2.06 (m, 3H, 3CH); 6,46 (s, 1H, 1CH); 7.29–7.33 (m, 2H, 2CH); 7.44–7.48 (m, 4H, 4CH); 7.71 (d, 2H, 2CH, J = 8.4 Hz); 7.95 (d, 2H, 2CH, J = 8.4 Hz); 8.22 (s, 1H, CH); 8.26 (d, 2H, 2 CH, J = 7.7 Hz); 12,34 (bs, 1H, NH). ^13^C NMR (176 MHz, DMSO-d_6_), δ (ppm): 28.34 (3C); 36.37 (3C); 36.75 (3C); 41.69 (C); 100.60 (C); 110.22 (2C); 120.75 (2C); 121.05 (2C); 123.38 (2C); 126.81 (2C); 127.34 (2C); 128.38 (2C); 133.87 (C); 137.99 (C); 140.35 (2C); 162.45 (C); 168.45 (C). ESI-HRMS (*m*/*z*) calculated for C_32_H_31_N_4_S: 503.2269 [M+H]^+^. Found: 503.2273 [M+H]^+^.

(*E*)-4-(2-(2-(4-(9*H*-carbazol-9-yl)benzylidene)hydrazinyl)thiazol-4-yl)benzonitrile (**K2**). Yield: 0.46 g, 97%, (dichloromethane/methanol (95:5), R_f_ = 0.79); mp 240–242 °C. ^1^H NMR (700 MHz, DMSO-d_6_), δ (ppm): 7.30–7.34 (m, 2H, 2CH); 7.42–7.49 (m, 4H, 4CH); 7.69 (s, 1H, 1CH); 7.71 (d, 2H, 2CH, J = 8.4 Hz); 7.89 (d, 2H, 2CH, J = 8.4 Hz); 7.96 (d, 2H, 2CH, J = 8.4 Hz); 8.06 (d, 2H, 2CH, J = 8.4 Hz); 8.21 (s, 1H, 1CH); 8.27 (d, 2H, 2CH, J = 7.7 Hz); 12.41 (bs, 1H, NH). ^13^C NMR (176 MHz, DMSO-d_6_), δ (ppm): 108.22 (C); 110.12 (C); 110.23 (C); 119.46 (C); 120.76 (2C); 121.06 (2C); 123.38 (2C); 126.63 (2C); 126.82 (2C); 127.39 (2C); 128.43 (2C); 130.20 (2C); 133.88 (C); 138.01 (C); 139.25 (C); 140.40 (2C); 141.30 (C); 149.39 (C); 168.98 (C). ESI-HRMS (*m*/*z*) calculated for C_29_H_20_N_5_S: 470.1439 [M+H]^+^. Found: 470.1433 [M+H]^+^.

(*E*)-2-(2-(4-(9*H*-carbazol-9-yl)benzylidene)hydrazinyl)-4-(4-nitrophenyl)thiazole (**K3**). Yield: 0.44 g, 90%, (dichloromethane/methanol (95:5), R_f_ = 0.73); mp 239–242 °C. ^1^H NMR (700 MHz, DMSO-d_6_), δ (ppm): 7.28–7.33 (m, 2H, 2CH); 7.43–7.49 (m, 4H, 4 CH); 7.71 (d, 2H, 2CH, J = 8.8 Hz); 7.75 (s, 1H, 1CH); 7.96 (d, 2H, 2CH, J = 8.8 Hz); 8.11 (d, 2H, 2CH, J = 8.8 Hz); 8.19 (s, 1H, 1CH); 8.25 (d, 2H, 2CH, J = 7.6 Hz); 8.27 (d, 2H, 2 CH, J = 8.6 Hz); 12.42 (bs, 1H, NH). ^13^C NMR (176 MHz, DMSO-d_6_), δ (ppm): 109.25 (C); 110.22 (2C); 120.05 (2C); 123.37 (2C); 124.59 (2C); 126.83 (2C); 127.35 (2C); 128.42 (2C); 133.81 (C); 138.02 (C); 140.35 (2C); 141.10 (C); 141.39 (C); 146.71 (C); 149.05 (C); 169.09 (C). ESI-HRMS (*m*/*z*) calculated for C_28_H_20_N_5_O_2_S: 490.1338 [M+H]^+^. Found: 490.1338 [M+H]^+^.

### 3.2. Mushroom Tyrosinase Inhibition Assay

The mushroom tyrosinase (Sigma-Aldrich, St. Louis, MO, USA) inhibition was performed following previously reported methods [53,54]. All the assays were carried out with solutions containing phosphate buffer (50 mM, pH 6.8), L-DOPA (0.17 mM), EDTA (0.022 mM), tyrosinase (50–100 units), and varying concentrations of tested carbazole, which were performed in triplicate at room temperature. The inhibitor solutions were prepared in DMSO with an initial concentration of 1 mM. Different aliquots were added to the solution containing buffers L-DOPA and EDTA, with the enzyme being added at the end. Formation of dopachromone was determined by monitoring the absorbance at 475 nm with a T60U spectrophotometer (PG Instruments, Leicestershire, UK) equipped with quartz cells of 1 cm path length. Kojic acid and ascorbic acid were used as reference inhibitors with an initial concentration of 1 mM. The IC_50_ values were calculated from the equation generated by exponential fit of the experimental data. The effectiveness of inhibition was expressed for the investigated compounds as the percentage of concentration necessary to achieve 50% inhibition (IC50), calculated using the following equation:

% of Inhibition = {[(B_30_ − B_0_) − (A_30_ − A_0_)]/(B_30_ − B_0_)} × 100

where B_0_ = absorbance of L-DOPA + tyrosinase at t = 0 min, B_30_ = absorbance of L-DOPA + tyrosinase at t = 30 min, A_0_ = absorbance of L-DOPA + tyrosinase + inhibitor at t = 0 min, and A_30_ = absorbance of L-DOPA + tyrosinase + inhibitor at t = 30 min.

### 3.3. Kinetic Analysis of the Inhibition of Tyrosinase

A series of experiments were performed to determine the inhibition kinetics of tested carbazoles by following the already reported method [9,55]. The inhibitor concentrations for tested carbazoles were 0.05 and 0.1 mM. Substrate L-DOPA concentration was between 0.1 and 0.25 mM in all kinetic studies. Maximal initial velocity was determined from the initial linear portion of absorbance up to ten minutes after addition of enzyme. The inhibition type of the enzyme, Michaelis constant (K_m_) and maximal velicity (V_max_), were determined by Lineweaver–Burk plots of inverse of velocities (1/V) versus inverse of substrate concentration 1/[L-DOPA] mM^−1^.

### 3.4. Thermogravimetric Analysis

Thermogravimetric analysis was performed on Perkin Elmer Thermogravimetric Analyzer Pyris 1 TGA at a heating rate of 15 °C/min under nitrogen. The electronic spectra were measured on a ThermoScientific Evolution 220 UV/Vis spectrometer. Photoluminescence emission spectra were acquired using Hitachi Fluorescence Spectrophotometer F-7100 [19].

### 3.5. Cyclic Voltammetry Analysis

Cyclic voltammetry measurements were carried out using a classic three-electrode system. A Pt wire was used as the working electrode, a Pt spiral was used as the counter electrode, and a leakless Ag/AgCl electrode (eDAQ) was used as the reference electrode. The potential was calibrated using ferrocene. Tetrabutylammonium tetrafluoroborate Bu_4_NBF_4_ (TCI) in CH_2_Cl_2_ (99.8% Sigma Aldrich, HPLC grade) with a concentration of 0.1 mol∙dm^−3^ was used as the supporting electrolyte. Solutions with **K1**, **K2**, and **K3** concentrations of 1 mmol∙dm^−3^ were purged with argon before measurements. The measurements were carried out with a scan rate of 50 mV∙s^−1^ using Autolab PGSTAT 100N (Metrohm Autolab B.V., Utrecht, The Netherlands) [20].

## 4. Conclusions

The three carbazole–thiazoles were synthesized and their ability to inhibit tyrosinase and the mechanism of its inhibition were studied. The physicochemical investigations were performed and supported with the DFT calculations. Finally, the prototype Concanavalin A-carbazole supramolecular complexes were investigated using molecular docking. Based on our research, it can be concluded that the investigated compounds **K1** and **K3** demonstrated a higher ability to inhibit tyrosinase than kojic acid. Results of our theoretical predictions of ability of compounds **K1**–**K3** to form stable supramolecular complexes with model proteins, including ConA and has, further enhances their potential applicability in bioimaging. The results of our theoretical predictions of the ability of compounds **K1**–**K3** to form stable supramolecular complexes with model proteins, including ConA and HSA, allow us to hypothesize that such a supramolecular complexes, upon entering the cell, will be able to release appropriate dyes due to ConA specificity for strong binding to polysaccharides containing α-glucose, mannose, glucosamine, and α-N-acetylglucosamine. Upon entering in the cell, the investigated compounds, particularly compound **K3** due to its strong sensitivity to environmental polarity, efficient charge transfer, and notable fluorescence properties, can be used as a promising candidate for fluorescence-based imaging. Additionally, it will also have higher possibility to inhibit tyrosinase. However, further in vivo and cellular validation studies are needed. Therefore, the search for new carbazoles with more favorable chemical properties and greater biological activity is the subject of ongoing research in the fields of medicinal chemistry and materials engineering.

## Figures and Tables

**Table 1:** Tyrosinase inhibitory activity of tested carbazoles compared to standard inhibitors. Determined inhibition type, maximum velocity (Vmax), and inhibition constant (K_m_) of **K1**–**K3** carbazoles.

Compound	IC_50_ ± SD [µM]	Inhibitory Mechanism	V_max_ [mM/min]	K_m_ [mM/min]
**K1**	59.36 ± 1.427	Mixed	0.2608	0.655
**K2**	81.99 ± 3.387	Mixed	0.4078	0.314
**K3**	45.95 ± 3.152	Mixed	0.4718	0.751
Ascorbic acid	386.50 ± 11.958	-	-	-
Kojic acid	72.27 ± 3.145	-	-	-

**Table 2:** Thermal properties of **K1**–**K3**.

Compound	T_5_ ^a^ [°C]	T_10_ ^a^ [°C]	T_max_ ^b^ [°C]
**K1**	222	279	301, 468
**K2**	231	252	313
**K3**	244	271	298, 668

^a^ T_5_ and T_10_ are temperature at 5% and 10% weight loss, respectively. ^b^ Temperature of maximum decomposition rate.

**Table 3:** Optical characterization of **K1**–**K3**.

Compound		Absorption (λ_abs_) [nm]	ε [M^−1^ cm^−1^]	Emission (λ_em_) [nm]	Stokes Shift ^a^ [cm^−1^]
**K1**	Toluene	362 340 326 291	18,949 15,630 12,532 12,910	416	54
	CHCl_3_	363 340 326 291	16,942 13,099 11,245 10,984	439	75
	THF	357 291	20,853 12,831	456	100
	EtOH	355 291	26,895 16,018	468	115
	DMSO	362 293	22,354 14,292	485	123
**K2**	Toluene	362 340 328 293	10,289 10,811 10,289 9859	409; 432	47; 70
	CHCl_3_	367 326 291 258	20,657 20,988 21,829 33,111	451	84
	THF	342 291 281 257	21,634 14,393 16,167 24,112	439	97
	EtOH	342 291 254	28,198 18,266 30,235	441	99
	DMSO	345 293 281	28,389 19,278 21,595	462	117
**K3**	Toluene	-	-	-	-
	CHCl_3_	361 291	26,666 17,688	444	83
	THF	358 291 283	27,103 16,416 14,577	nd	-
	EtOH	356 291 281	14,913 9712 8671	447	92
	DMSO	362 293	35,033 22,045	475	113

^a^ λ_em_—λ_abs_ for solutions; nd—not detected.

## Data Availability

The data presented in this study are available on request from the corresponding author. Compounds will be provided upon reasonable scientific request.

## Supplementary Materials

The supporting information can be downloaded at: https://www.mdpi.com/article/10.3390/ijms26167945/s1.

## Abbreviations

The following abbreviations are used in this manuscript:

L-DOPA	L-3,4-dihydroxyphenylalanine
ESI	electrospray ionization
HRMS	high-resolution mass spectrometry
IC_50_	Half-maximal inhibitory concentration
SD	standard deviation
V_max_	maximum velocity
K_m_	inhibition constant
TGA	thermogravimetric analysis
THF	tetrahydrofuran
EtOH	ethanol
DMSO	dimethyl sulfoxide
CHCl_3_	chloroform
CT	charge transfer
NBO	natural bond orbital
LUMO	lowest unoccupied molecular orbital
HOMO	highest occupied molecular orbital
MEP	molecular electrostatic potential
NLO	nonlinear optical materials
ConA	concanavalin A
HSA	human serum albumin
TDDFT	time-dependent density-functional theory
PBE0	Perdew–Burke–Ernzerhof hybrid functional
