# Supplementary material for: Novel Carbazole–Thiazole Conjugates: Synthesis and Biophysical Characterization
Source: Int J Mol Sci. 2025 Aug 18;26(16):7945. doi: 10.3390/ijms26167945 (PMC12386811; doi:10.3390/ijms26167945)
Supplement: Supplementary file 1 [file ijms-26-07945-s001.zip › ijms-3756532-supplementary.pdf]

SUPLEMENTARY MATERIALS

Novel Carbazole–Thiazole Conjugates: Synthesis and Biophysical Characterization

Beata Donarska [1], Klaudia Seklecka [1], Joanna Cytarska [1], Katarzyna Piechowska [1], Przemyslaw Ledwon [2], Sławomir Kula [3], Przemysław Krawczyk [4], Angelika Baranowska-Łączkowska [5,*] and Krzysztof Z. Łączkowski [1,*]

1 Department of Chemical Technology and Pharmaceuticals, Faculty of Pharmacy, Collegium Medicum, Nicolaus Copernicus University, Jurasza 2, 85-089 Bydgoszcz, Poland; beata.donarska@cm.umk.pl (B.D.); imaginowa@o2.pl (K.S.); cytar@cm.umk.pl (J.C.); kpiechowska@cm.umk.pl (K.P.)
2 Department of Physical Chemistry and Technology of Polymers, Faculty of Chemistry, Silesian University of Technology, Strzody 9, 44-100 Gliwice, Poland; przemyslaw.ledwon@polsl.pl
3 Institute of Chemistry, Faculty of Science and Technology, University of Silesia, Szkolna 9 St., 40-007 Katowice, Poland; slawomir.kula@us.edu.pl
4 Department of Physical Chemistry, Faculty of Pharmacy, Collegium Medicum, Nicolaus Copernicus University, Kurpińskiego 5, 85-950 Bydgoszcz, Poland; przemekk@cm.umk.pl
5 Faculty of Physics, Kazimierz Wielki University, Powstańców Wielkopolskich 2, 85-090 Bydgoszcz, Poland
* Correspondence: anxela@ukw.edu.pl (A.B.-Ł.); krzysztof.laczkowski@cm.umk.pl (K.Z.Ł.)

Contents:

1. UV-Vis absorption measurements

2. Computational details

Figure S1. Absorption (a) and emission (b) spectra for K2.

Figure S2. Absorption (a) and emission (b) spectra for K3.

Figure S3. Molecular structure of the studied carbazole derivatives with indicated positions of carbon atoms C38 and C43 relevant to NBO analysis.

Figure S4. Visualization of HOMO and LUMO orbitals for carbazole K1-K3.

Figure S5. Molecular electrostatic potential (MEP) surfaces of carbazole derivatives K1-K3.

Figure S6. Electron density difference maps (Δρ(r)) upon photoexcitation for K1-K3.

Table S1. Occupancy (e), energy (a.u.) and polarity (%) of natural bond orbitals (NBOs) and hybrids calculated for investigated complexes (C38-C43).

Table S2. The frontier orbital energies in selected solvents. All values are given in eV.

Table S3. Charge-transfer descriptors: the amount of transferred charge (qCT) and charge-transfer distance (DCT) for K1-K3 in various solvents.

Table S4. Calculated free energy of solvation (ΔGsolv in kcal/mol) for tested carbazole derivatives in various solvents of increasing polarity.

Table S5. The theoretical vertical and cLR corrected excitation energies in nm.

Table S6. Calculated values of dipole moments (in D) for the ground and CT excited state.

Table S7. The theoretical de-excitation energies in nm determined using PBE0 functional.

Table S8. Nonlinear optical properties. All values are given in [a.u.].
Table S9. Binding free energies (ΔG_b, kcal/mol) obtained during AutoDock simulations with Conconavalin A.
Table S10. Binding free energies (ΔG_b, kcal/mol) obtained during AutoDock simulations with Human Serum Albuminum.
^{1}H, ^{13}C NMR and ESI-HRMS spectra of compounds K1-K3.

1. UV-Vis absorption measurements

Steady-state absorption and emission spectra were recorded on a Shimadzu UV-Vis Multispec-1501 spectrophotometer and a Hitachi F-7100 spectrophotometer, respectively. The fluorescence quantum yields for the dyes were calculated using equation.

$$\phi_s = \phi_{ref} \frac{I_s A_{ref}}{I_{ref} A_s} \cdot \frac{n_s^2}{n_{ref}^2}$$

where: ϕ_{ref} is the fluorescence quantum yield of reference (Coumarin 1; $\phi_{ref} = 0.64$ [27]) sample in ethanol, A_s and A_{ref} are the absorbances of the dye and reference samples at the excitation wavelengths (A≈0.1 at 366 nm), I_s and I_{ref} are the integrated emission intensity for the compound tested and reference samples, n_s and n_{ref} are the refractive indices of the solvents used for the compound tested and the reference, respectively.

The fluorescence lifetimes were measured using an Edinburgh Instruments single-photon counting system (FLS920P Spectrometers). The apparatus utilizes a picosecond diode laser for the excitation generating pulses of about 55 ps at 375 nm. The dyes were studied at dilute solution (A≈0.1 in a 10 mm cell). The fluorescence decays were usually fitted to double-exponential functions. The average lifetime, τ_{av} is calculated as

$$\tau_{av} = \frac{\Sigma \tau_i \alpha_i}{\Sigma \alpha_i}$$

were α_i and τ_i are the amplitudes and lifetimes.

2. Computational details

The geometries of all studied molecules in both ground and excited states were optimized using the density functional theory (DFT) approach implemented in the Gaussian 16 software package [28], employing the PBE0 functional with the 6-311++G(d,p) basis set. To confirm that the obtained structures represent true minima on the potential energy surface,

vibrational frequency calculations were carried out through Hessian analysis. Electronic properties were examined by computing vertical absorption and emission spectra using time-dependent DFT (TDDFT/PBE0) [29], incorporating the state-specific corrected linear response method (SS-cLR). All spectroscopic computations were performed using the hybrid PBE0 functional [30–35]. To accurately account for solvent effects on fluorescence, the ground-state geometry was calculated using a non-equilibrium solvation approach [36,37].

Dipole moments in the excited state (μ_{CT}) and the polarity of the charge-transfer (CT) state were calculated by numerical differentiation of excitation energies (*E*) under an external electric field *F* of 0.001 a.u. [38]:

$$\Delta\mu_i = \mu_i^{CT} - \mu_i^{GS} = \frac{E^{CT}(+F_i) - E^{CT}(-F_i)}{-2F_i} - \frac{E^{GS}(+F_i) - E^{GS}(-F_i)}{-2F_i}$$

where F_i is the electric field component along Cartesian axis i, and μ_i is the i-th component of the dipole moment vector.

Density difference maps were generated at the PBE0/6-311++G(d,p) level with a contour threshold of 0.02 a.u. In these maps, regions of electron depletion and accumulation upon excitation are shown in blue and purple, respectively. Charge-transfer parameters, including the charge-transfer distance (D_{CT}) and the amount of transferred charge (q_{CT}), were calculated according to the method described by Le Bahers [39]. Solvent effects on linear and nonlinear optical properties were modeled using the integral equation formalism of the polarizable continuum model (IEF–PCM) [40,41].

The isotropic average polarizability (α) and first-order hyperpolarizability (β) were obtained from Gaussian 16 outputs and computed as follows:

$$\langle\alpha\rangle = \frac{\alpha_{xx}+\alpha_{yy}+\alpha_{zz}}{3}$$

$$\beta_{vec} = \sum_{i=x,y,z} \frac{\mu_i\beta_i}{|\mu|}$$

where $\beta_i (i = x, y, z)$ is given by $\beta_i = \left(\frac{1}{3}\right)\sum_{j=x,y,z}\left(\beta_{ijj} + \beta_{jij} + \beta_{jji}\right)$

Two-photon absorption (TPA) was modeled as a function of photon energy dissipation under single-beam excitation, where the transition rate is doubled. The cross-section for the degenerate two-photon absorption process is expressed as [42,43]:

$$\sigma_{OF}^{(2)} = \frac{8\pi^3\alpha^2\eta^3}{e^4} \cdot \frac{\omega^2 g(\omega)}{\Gamma_F/2} \langle\delta_{OF}\rangle$$

where α is a fine structure constant, ω is the frequency of absorbed photons (assuming one source of photons), Γ_F is the broadening of the final state (F) due to its finite lifetime and g(ω) provides the spectral line profile, which often is assumed to be a δ-function and $\langle \delta^{OF} \rangle$ is the two-photon transition probability for the transition from the ground state to a final state. For an isotropic medium using linearly polarized light, this probability is given by [44]:

$$\langle \delta_{OF} \rangle = \frac{1}{15} \sum^{ij} \left[S_{OF}^{ii} \left(S_{OF}^{jj} \right)^* + 2 S_{OF}^{ij} \left(S_{OF}^{ij} \right)^* \right]$$

The second-order transition moment S_{OF}^{ij} is defined as:

$$S_{OF}^{ij}(\zeta_1, \zeta_2) = \frac{1}{\eta} \sum_K \left[\frac{\langle 0 | \zeta_1 \cdot \mu_i | K \rangle \langle K | \zeta_2 \cdot \mu_j | F \rangle}{\omega_\alpha - \omega_1} + \frac{\langle 0 | \zeta_2 \cdot \mu_i | K \rangle \langle K | \zeta_1 \cdot \mu_j | F \rangle}{\omega_\alpha - \omega_2} \right]$$

To describe the two-photon allowed transitions, the quadratic response theory [45,46] within the DFT framework was employed as implemented in the DALTON 2011 software [47,48], with solvation modeled via the self-consistent reaction field (SCRF) approach. All TPA calculations used the CAM-B3LYP functional and the 6-311++G(d,p) basis set.

Binding affinities of the compounds were evaluated using the united-atom scoring function in AutoDock Vina [49]. Molecular docking was conducted by targeting the active sites of two proteins: Concanavalin A (PDB ID: 2a7a) [50] and human serum albumin (HSA) [51]. The docking grid was set to enclose the amino group of lysine side chains, with a cubic grid box of 16 Å and a spacing of 1 Å. Ten independent docking runs were performed for each lysine residue, allowing for identification of binding sites with the highest affinity for each fluorophore.

Biological activity predictions were conducted using a combined approach based on the 3D/4D QSAR BiS/MC and CoCon algorithms [52].

Figure S1. Absorption (a) and emission (b) spectra for K2.

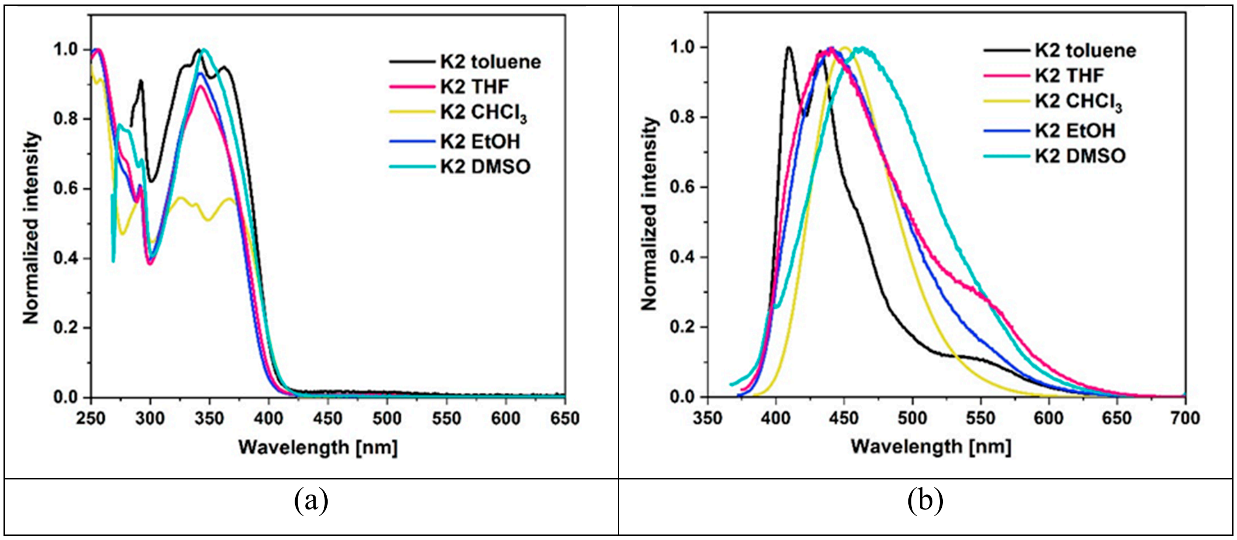

(a)	(b)

Figure S2. Absorption (a) and emission (b) spectra for K3.

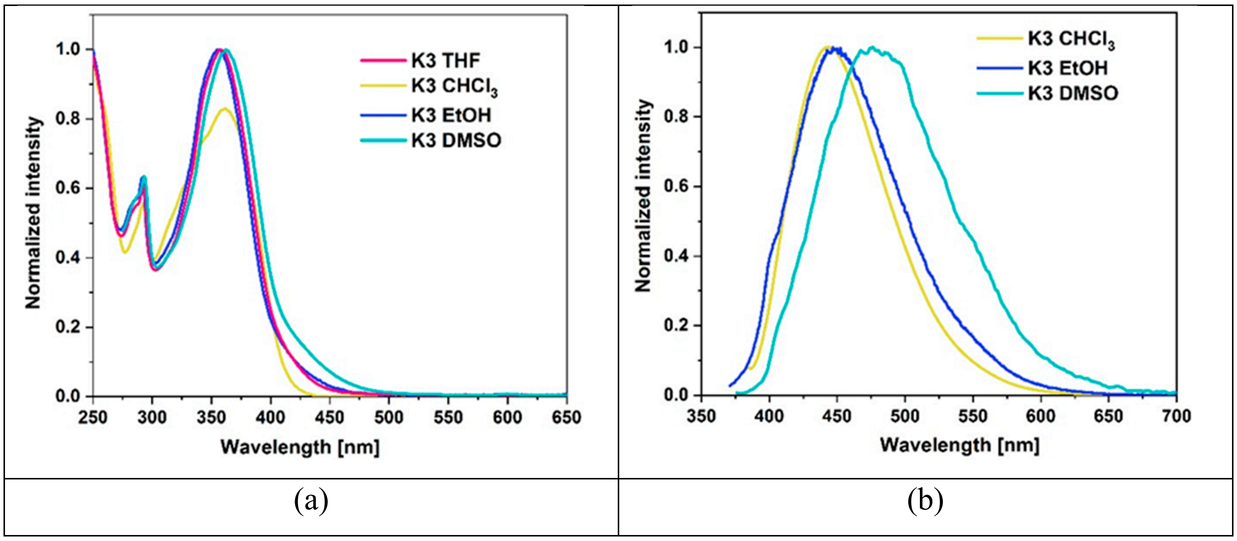

(a)	(b)

Figure S3. Molecular structure of the studied carbazole derivatives with indicated positions of carbon atoms C38 and C43 relevant to NBO analysis.

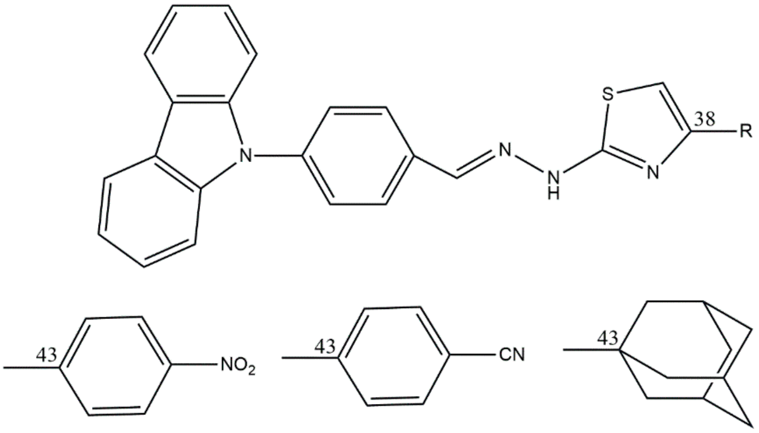

Figure S4. Visualization of HOMO and LUMO orbitals for carbazole K1-K3.

K1	K2	K3
LUMO		
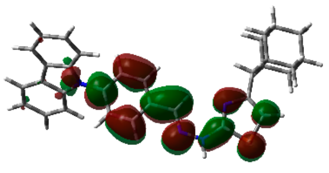	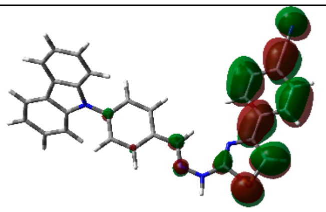	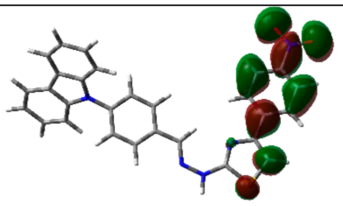
HOMO		
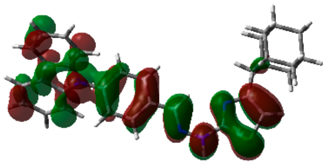	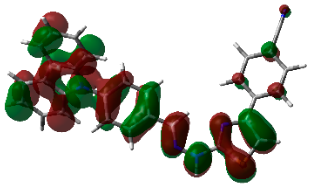	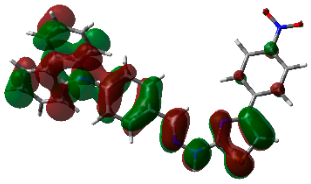

Figure S5. Molecular electrostatic potential (MEP) surfaces of carbazole derivatives K1-K3.

K1	K2	K3
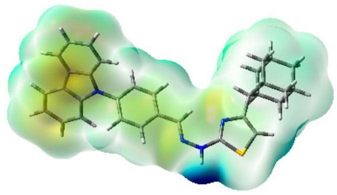	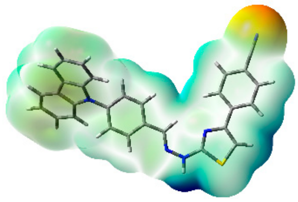	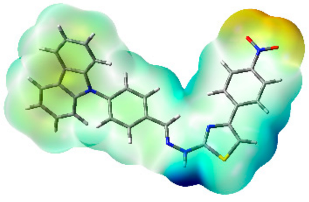

Figure S6. Electron density difference maps (Δρ(r)) upon photoexcitation for K1-K3.

K1	K2	K3
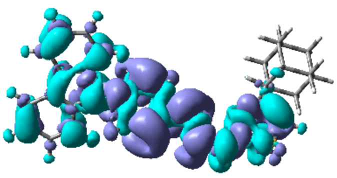	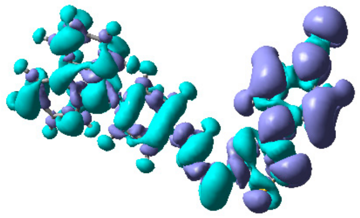	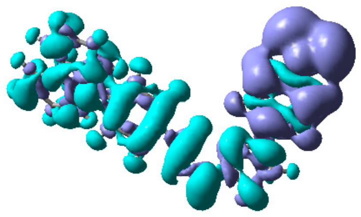

Table S1. Occupancy (e), energy (a.u.) and polarity (%) of natural bond orbitals (NBOs) and hybrids calculated for investigated complexes (C38-C43).

	NBO orbital	Orbital energy	Ocupancy		Charge	Polarity	NBO hybrid	Atomic orbitals
**K1**								
TOL	σ (C – C)	-0.64743	1.96735	C38	0.16061	50.61	$sp^{1.79}$	s(35.78%) p(64.20%)
				C43	-0.25439	49.39	$sp^{2.69}$	s(27.05%) p(72.89%)
TCM	σ (C – C)	-0.64744	1.96738	C38	0.15912	50.62	$sp^{1.79}$	s(35.79%) p(64.19%)
				C43	-0.25476	49.38	$sp^{2.70}$	s(27.04%) p (72.90%)
THF	σ (C – C)	-0.65033	1.96742	C38	0.15818	50.64	$sp^{1.79}$	s(35.81%) p(64.17%)
				C43	-0.25516	49.36	$sp^{2.70}$	s(27.03%) p(72.91%)
EtOH	σ (C – C)	-0.65042	1.96746	C38	0.15688	50.65	$sp^{1.79}$	s(35.83%) p(64.16%)
				C43	-0.25550	49.35	$sp^{2.70}$	s(27.03%) p(72.92%)
MeOH	σ (C – C)	-0.65043	1.96747	C38	0.15672	50.65	$sp^{1.79}$	s(35.83%) p(64.16%)
				C43	-0.25554	49.35	$sp^{2.70}$	s(27.03%) p(72.92%)
DMSO	σ (C – C)	-0.65045	1.96747	C38	0.15656	50.65	$sp^{1.79}$	s(35.83%) p(64.15%)
				C43	-0.25557	49.35	$sp^{2.70}$	s(27.03%) p(72.92%)
H_2O	σ (C – C)	-0.65048	1.96748	C38	0.15641	50.65	$sp^{1.79}$	s(35.83%) p(64.15%)
				C43	-0.25561	49.35	$sp^{2.70}$	s(27.02%) p(72.92%
**K2**								
TOL	σ (C – C)		1.96816	C38	0.12601	49.85	$sp^{1.82}$	s(35.46%) p(64.51%)
				C43	-0.05390	50.15	$sp^{2.14}$	s(31.85%) p(68.11%)
TCM	σ (C – C)	-0.69949	1.96804	C38	0.11928	49.77	$sp^{1.80}$	s(35.68%) p(64.29%)
				C43	-0.04939	50.23	$sp^{2.14}$	s(31.88%) p(68.08%)
THF	σ (C – C)	-0.69969	1.96816	C38	0.11760	49.76	$sp^{1.80}$	s(35.69%) p(64.28%)
				C43	-0.04910	50.24	$sp^{2.13}$	s(31.91%) p(68.06%)
EtOH	σ (C – C)	-0.69901	1.96824	C38	0.11529	49.75	$sp^{1.80}$	s(35.69%) p(64.28%)
				C43	-0.04875	50.25	$sp^{2.13}$	s(31.92%) p(68.04%)
MeOH	σ (C – C)		1.96824	C38	0.11503	49.75	$sp^{1.80}$	s(35.69%) p(64.28%)
				C43	-0.04872	50.25	$sp^{2.13}$	s(31.93%) p(68.04%)
DMSO	σ (C – C)	-0.69888	1.96825	C38	0.11476	49.75	$sp^{1.80}$	s(35.69%) p(64.28%)
				C43	-0.04870	50.25	$sp^{2.13}$	s(31.93%) p(68.04%)
H_2O	σ (C – C)	-0.69882	1.96826	C38	0.11452	49.75	$sp^{1.80}$	s(35.69%) p(64.28%)
				C43	-0.04868	50.25	$sp^{2.13}$	s(31.93%) p(68.04%)
**K3**								

TOL	σ (C – C)	-0.70553	1.96823	C38	0.12411	49.82	$sp^{1.82}$	s(35.43%) p(64.54%)
				C43	-0.04906	50.18	$sp^{2.13}$	s(31.89%) p(68.08%)
TCM	σ (C – C)	-0.70365	1.96838	C38	0.12052	49.80	$sp^{1.82}$	s(35.43%) p(64.54%)
				C43	-0.04714	50.20	$sp^{2.13}$	s(31.94%) p(68.02%)d
THF	σ (C – C)	-0.70283	1.96850	C38	0.11874	49.79	$sp^{1.82}$	s(35.45%) p(64.52%)
				C43	-0.04210	50.21	$sp^{2.13}$	s(31.96%) p(68.00%)
EtOH	σ (C – C)	-0.70255	1.96855	C38	0.11651	49.78	$sp^{1.82}$	s(35.44%) p(64.53%)
				C43	-0.04595	50.22	$sp^{2.12}$	s(32.00%) p(67.96%)
MeOH	σ (C – C)	-0.70256	1.96856	C38	0.11624	49.78	$sp^{1.82}$	s(35.44%) p(64.53%)
				C43	-0.04585	50.22	$sp^{2.12}$	s(32.01%) p(67.96%)
DMSO	σ (C – C)	-0.70260	1.96857	C38	0.11597	49.78	$sp^{1.82}$	s(35.44%) p(64.53%)
				C43	-0.04575	50.22	$sp^{2.12}$	s(32.01%) p(67.96%)
H_2O	σ (C – C)	-0.70226	1.96858	C38	0.11572	49.77	$sp^{1.82}$	s(35.44%) p(64.53%)
				C43	-0.04565	50.23	$sp^{2.12}$	s(32.01%) p(67.95%)

Table S2. The frontier orbital energies in selected solvents. All values are given in eV.

	E_{HOMO}	E_{LUMO}	ΔE_{GAP}	η chemical hardness	μ chemical potential	χ electro-negativity	σ chemical softnes	ω global electrophilicity	*pi*	*S*	ΔNmax
K1 Tol	-5.6999	-1.5945	4.1055	2.0527	-3.6472	3.6472	0.4872	3.2401	-3.6472	1.0264	1.7767
K1 TCM	-5.8194	-1.6230	4.1964	2.0982	-3.7212	3.7212	0.4766	3.2999	-3.7212	1.0491	1.7735
K1 THF	-5.7938	-1.6663	4.1275	2.0638	-3.7301	3.7301	0.4846	3.3709	-3.7301	1.0319	1.8074
K1 EtOH	-5.8170	-1.6913	4.1256	2.0628	-3.7541	3.7541	0.4848	3.4161	-3.7541	1.0314	1.8199
K1 MeOH	-5.8194	-1.6943	4.1251	2.0625	-3.7569	3.7569	0.4848	3.4215	-3.7569	1.0313	1.8215
K1 DMSO	-5.8221	-1.6973	4.1248	2.0624	-3.7597	3.7597	0.4849	3.4270	-3.7597	1.0312	1.8230
K1 H_2O	-5.8243	-1.7000	4.1243	2.0621	-3.7622	3.7622	0.4849	3.4319	-3.7622	1.0311	1.8244
K2 Tol	-5.8711	-2.0587	3.8124	1.9062	-3.9649	3.9649	0.5246	4.1236	-3.9649	0.9531	2.0800
K2 TCM	-5.8828	-2.0372	3.8456	1.9228	-3.9600	3.9600	0.5201	4.0779	-3.9600	0.9614	2.0595
K2 THF	-5.8869	-2.0356	3.8513	1.9257	-3.9612	3.9612	0.5193	4.0743	-3.9612	0.9628	2.0571
K2 EtOH	-5.8951	-2.0280	3.8671	1.9335	-3.9615	3.9615	0.5172	4.0583	-3.9615	0.9668	2.0488
K2 MeOH	-5.8978	-2.0261	3.8717	1.9359	-3.9619	3.9619	0.5166	4.0542	-3.9619	0.9679	2.0466
K2 DMSO	-5.8970	-2.0269	3.8701	1.9350	-3.9619	3.9619	0.5168	4.0560	-3.9619	0.9675	2.0475
K2 H_2O	-5.8978	-2.0261	3.8717	1.9359	-3.9619	3.9619	0.5166	4.0542	-3.9619	0.9679	2.0466
K3 Tol	-5.8844	-2.7927	3.0918	1.5459	-4.3386	4.3386	0.6469	6.0882	-4.3386	0.7729	2.8065
K3 TCM	-5.8978	-2.8324	3.0654	1.5327	-4.3651	4.3651	0.6525	6.2159	-4.3651	0.7663	2.8480
K3 THF	-5.9043	-2.8515	3.0528	1.5264	-4.3779	4.3779	0.6551	6.2780	-4.3779	0.7632	2.8681
K3 EtOH	-5.9136	-2.8795	3.0341	1.5170	-4.3965	4.3965	0.6592	6.3708	-4.3965	0.7585	2.8981
K3 MeOH	-5.9144	-2.8825	3.0319	1.5159	-4.3984	4.3984	0.6597	6.3809	-4.3984	0.7580	2.9014
K3 DMSO	-5.9155	-2.8855	3.0300	1.5150	-4.4005	4.4005	0.6601	6.3909	-4.4005	0.7575	2.9046
K3 H_2O	-5.9163	-2.8885	3.0278	1.5139	-4.4024	4.4024	0.6605	6.4010	-4.4024	0.7570	2.9080

Table S3. Charge-transfer descriptors: the amount of transferred charge (q_{CT}) and charge-transfer distance (D_{CT}) for K1-K3 in various solvents.

	K1		K2		K3	
	q_{CT}	D_{CT}	q_{CT}	D_{CT}	q_{CT}	D_{CT}
Tol	0.663	2.300	0.767	5.140	1.169	6.457
TCM	0.641	1.903	0.724	5.317	1.117	6.417
THF	0.630	1.695	0.728	5.636	1.096	6.398
EtOH	0.624	1.457	0.767	5.829	1.073	6.346
MeOH	0.624	1.461	0.789	5.857	1.071	6.347
DMSO	0.622	1.412	0.792	5.851	1.069	6.341
H_2O	0.622	1.392	0.806	5.822	1.067	6.323

Table S4. Calculated free energy of solvation (ΔG_{solv} in kcal/mol) for tested carbazole derivatives in various solvents of increasing polarity.

	K1	K2	K3
	ΔG_{solv}		
Tol	-26.35	-24.31	-22.44
TCM	-27.45	-29.67	-27.50
THF	-24.29	-24.01	-23.72
EtOH	-24.08	-23.06	-21.78
MeOH	-23.15	-22.14	-20.61
DMSO	-20.72	-21.14	-20.55
H_2O	-10.88	-14.67	-14.06

Table S5. The theoretical vertical and cLR corrected excitation energies in nm.

	K1			K2			K3		
	λ_{abs}^{vert}	f	λ_{abs}^{cLR}	λ_{abs}^{vert}	f	λ_{abs}^{cLR}	λ_{abs}^{vert}	f	λ_{abs}^{cLR}
Tol	362.61	0.9769	362.18	362.95	0.7223	361.44	370.1	0.7924	365.13
TCM	363.53	1.0103	361.98	368.96	0.7879	356.06	366.56	0.8434	363.81
THF	360.73	1.0401	360.93	351.30	0.7431	356.64	365.00	0.8603	361.88
EtOH	360.20	1.0486	360.31	350.27	0.7071	358.77	361.20	0.8834	360.21

MeOH	360.78	1.0421	360.07	359.02	0.7404	359.33	362.73	0.8778	359.76
DMSO	362.91	1.0663	360.62	357.74	0.7269	359.84	363.53	0.9057	365.39
H_2O	360.87	1.0472	360.07	356.08	0.7800	360.71	362.55	0.8862	358.68

Table S6. Calculated values of dipole moments (in D) for the ground and CT excited state.

	K1		K2		K3	
	μ_{GS}	μ_{CT}	μ_{GS}	μ_{CT}	μ_{GS}	μ_{CT}
Tol	4.21	6.34	7.32	19.38	7.66	35.21
TCM	4.45	5.92	7.89	20.60	8.32	36.29
THF	4.52	7.06	8.14	19.22	8.62	37.17
EtOH	4.67	8.15	8.47	19.15	9.03	37.03
MeOH	4.69	8.39	8.51	19.28	9.07	37.06
DMSO	4.71	8.15	8.55	18.84	9.12	36.91
H_2O	4.72	8.59	8.58	19.21	9.16	37.02

Table S7. The theoretical de-excitation energies in nm determined using PBE0 functional.

	K1	K2	K3
	λ_{em}		
Tol	425.14	434.81	432.93
TCM	442.30	441.63	443.03
THF	463.80	445.39	448.13
EtOH	470.95	448.59	451.61
MeOH	475.83	454.52	456.92
DMSO	487.83	466.25	477.18
H_2O	490.37	471.34	477.47

Table S8. Nonlinear optical properties. All values are given in [a.u.].

	K1		K2		K3	
	α	β	α	β	α	β
Tol	526.29	890.71	520.17	1000.44	525.08	3503.14
TCM	567.56	1343.69	561.38	1733.78	569.03	5378.44
THF	588.71	1436.82	581.53	2117.05	590.20	6436.17
EtOH	618.58	1768.07	610.52	2750.55	621.61	8168.59
MeOH	622.65	1805.59	613.86	2826.27	625.24	8379.51
DMSO	625.51	1843.68	617.12	2902.13	628.88	8592.38
H_2O	628.79	1877.40	620.34	2974.45	632.32	8796.30

Table S9. Binding free energies (ΔG_b, kcal/mol) obtained during AutoDock simulations with Conconavalin A.

LYS	**K1**	**K2**	**K3**
30	-3.3	-3.4	-3.2
35	-4.8	-4.7	-4.5
36	-5.3	-4.6	-4.9
39	-4.1	-3.6	-3.5
46	-5.4	-5.4	-5.3
59	-4.8	-4.4	-4.7
101	-5.4	-5.2	-5.1
114	-5.0	-5.1	-4.8
116	-6.3	-5.2	-5.1
135	-4.4	-3.9	-4.1
138	-3.9	-4.2	-3.7
200	-3.7	-3.9	-3.7
Ter	-3.6	-3.2	-3.2

Table S10. Binding free energies (ΔG_b, kcal/mol) obtained during AutoDock simulations with Human Serum Albuminum.

CYS	**K1**	**K2**	**K3**
34	-4.6	-.35	-.39
53	-3.9	-4.7	-4.5
62	-4.0	-4.7	-4.4
75	-6.1	-5.5	-5.8
90	-4.1	-2.3	-3.6
91	-5.4	-5.2	-5.1
101	-5.4	-5.1	-5.7
124	-5.4	-5.2	-5.4
168	-4.8	-5.3	-4.9
169	-4.8	-4.1	-4.5
177	-5.7	-5.3	-5.3

200	-1.6	-4.1	-3.0
245	-0.8	-0.2	-0.7
246	-1.2	-1.8	-4.9
253	-1.1	-1.3	-1.5
265	-2.9	-0.9	-0.4
278	-5.9	-4.1	-4.5
279	-4.7	-4.6	-5.1
289	-0.9	-4.1	-4.3
316	-6.5	-6.7	-6.5
360	-5.8	-4.6	-4.7
361	-5.4	-5.8	-5.5
369	-5.4	-5.0	-4.9
392	-1.7	-0.8	-1.6
437	-6.6	-6.4	-6.4
438	-7.1	-6.1	-6.2
444	-10.3	-9.0	-9.0
461	-1.9	-1.5	-2.1
476	-5.4	-4.1	-4.9
477	-2.2	-4.7	-2.5
487	-6.0	-6.1	-6.2
514	-5.6	-5.4	-5.3
558	-3.3	-3.2	-3.0
559	-4.8	-4.1	-4.2
567	-0.4	-0.4	-0.4

^{1}H, ^{13}C NMR and ESI-HRMS spectra of compounds K1-K3.

Compound K1

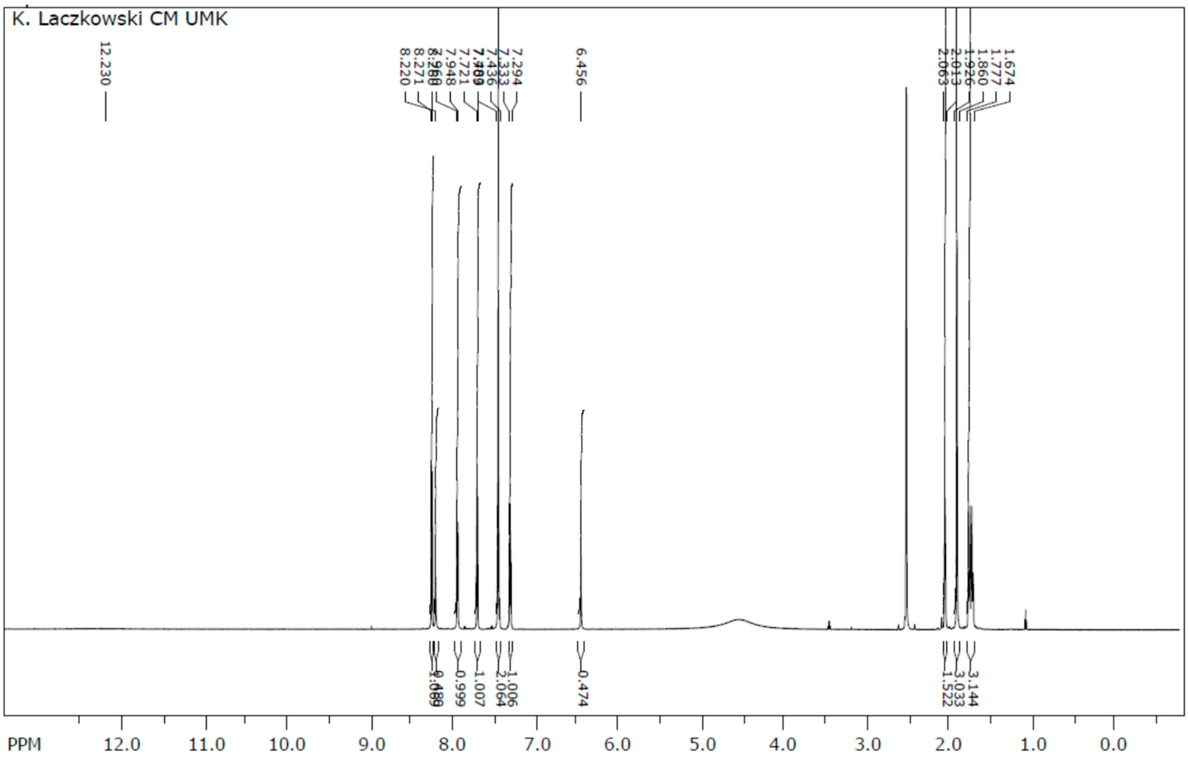

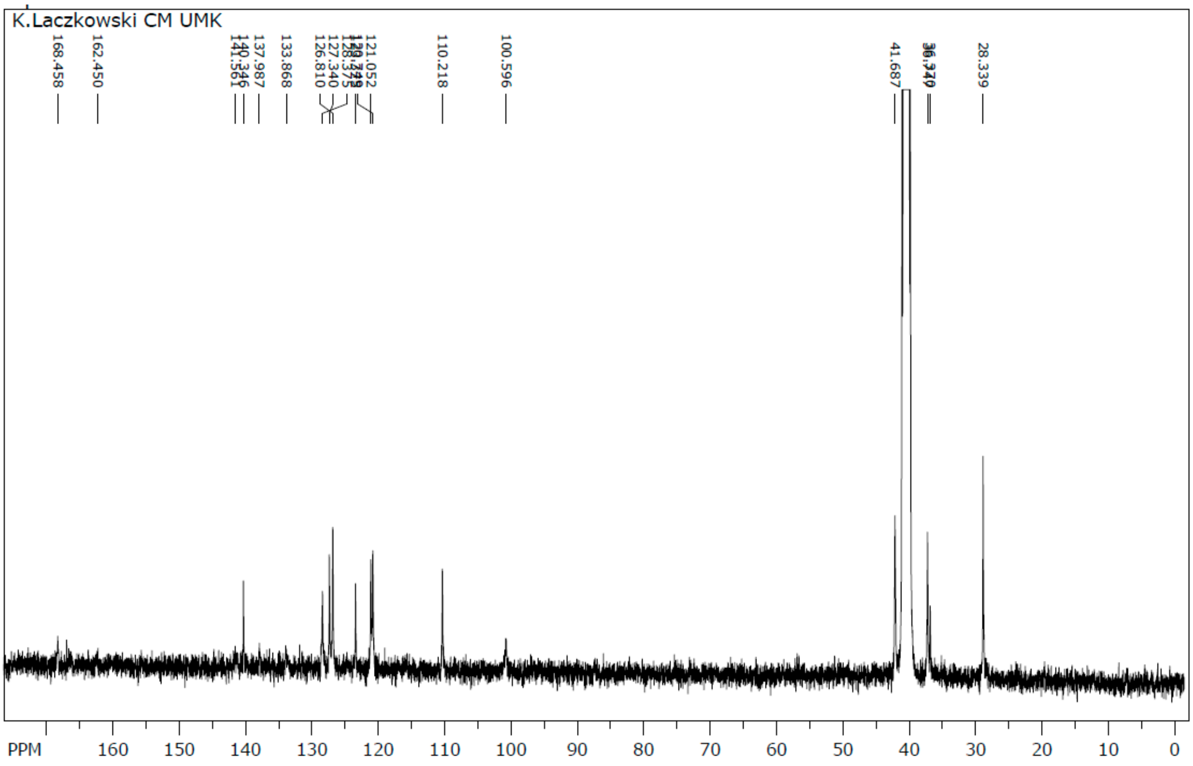

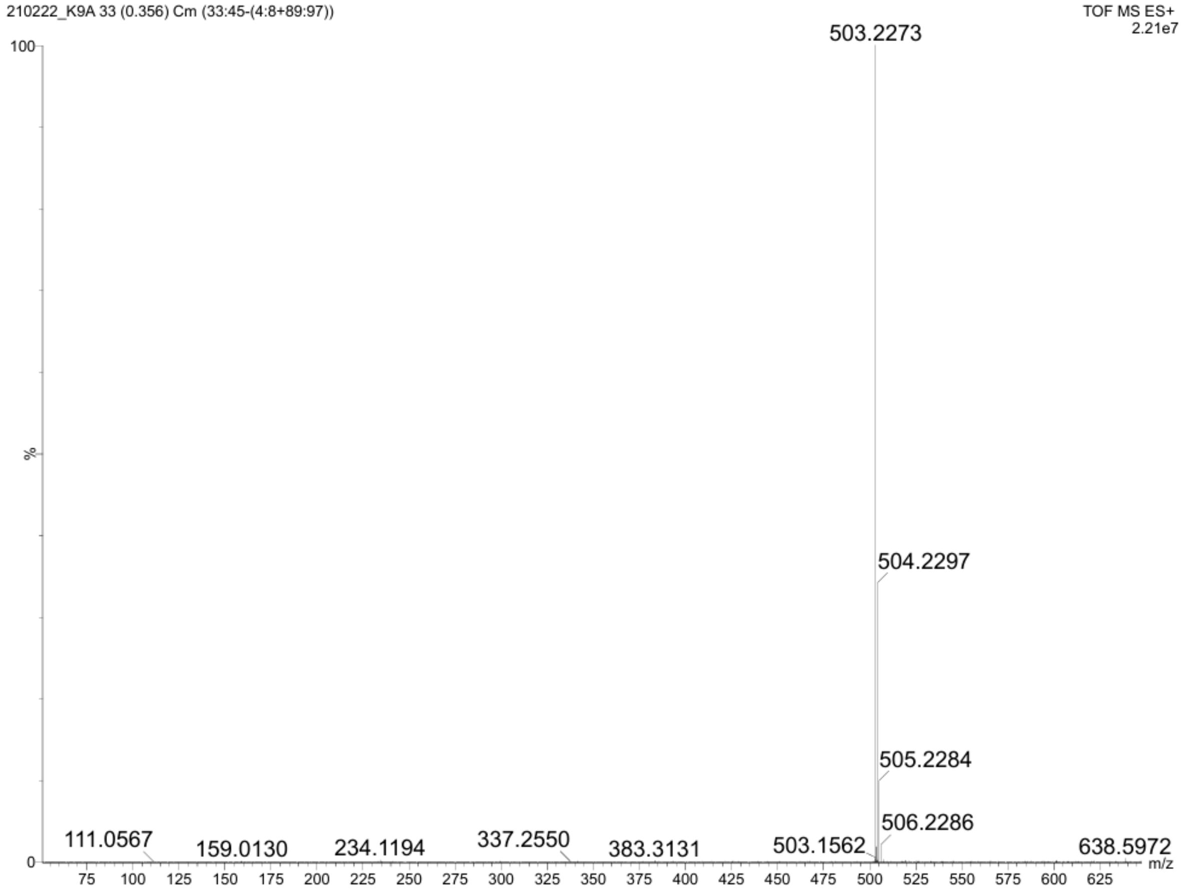

210222_K9A 33 (0.356) Cm (33:45-(4:8+89:97))
TOF MS ES+
2.21e7
503.2273
504.2297
505.2284
506.2286
111.0567
159.0130
234.1194
337.2550
383.3131
503.1562
638.5972
m/z
%

Compound K2

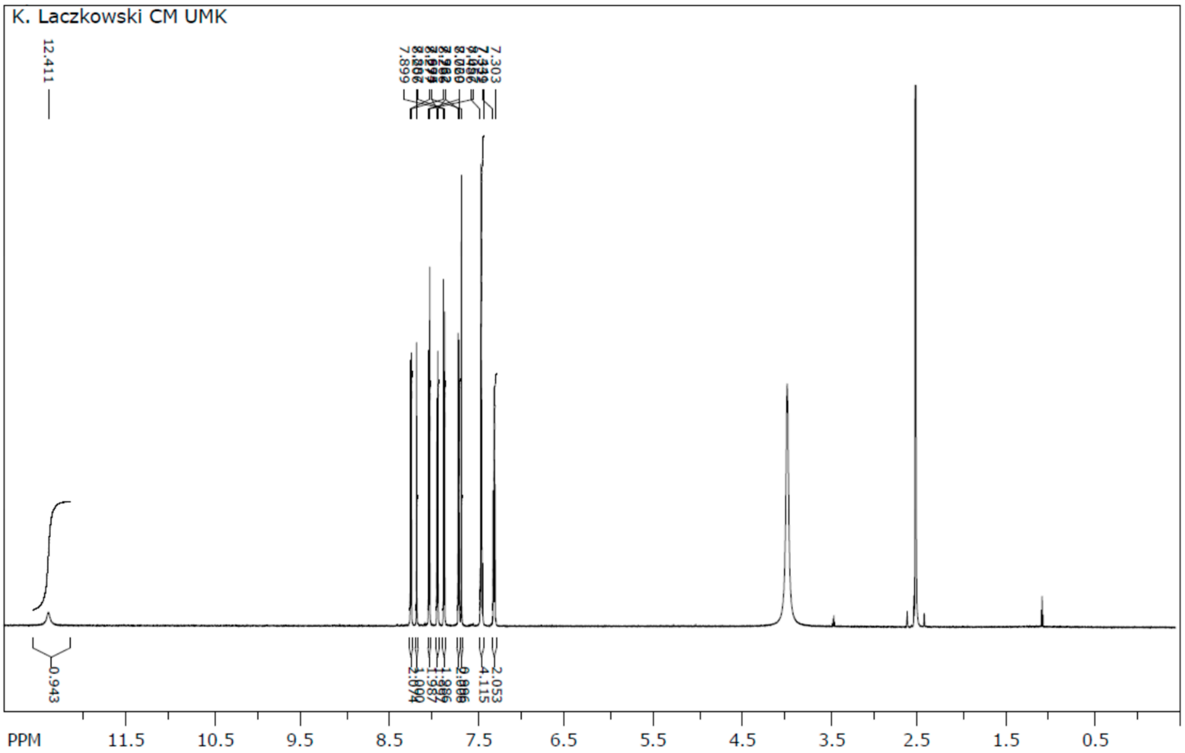

K. Laczkowski CM UMK
12.411
PPM

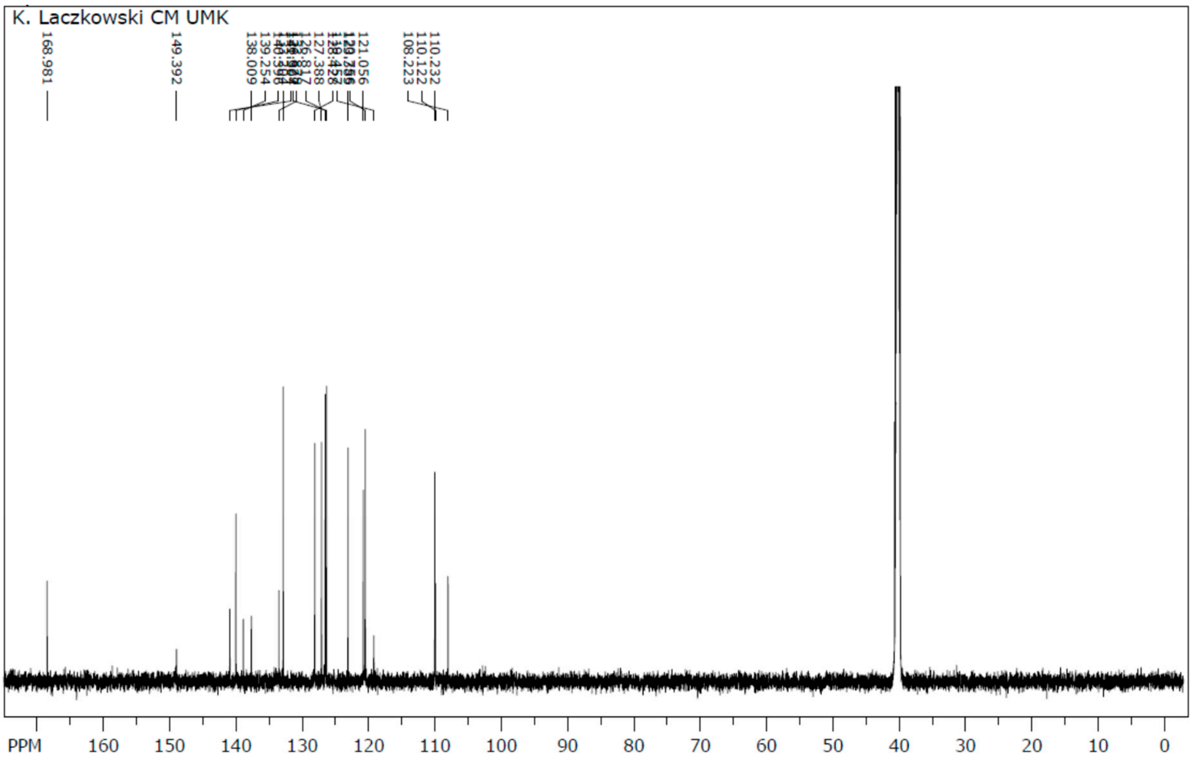
K. Laczkowski CM UMK
168.981
149.392
139.254
138.009
127.388
121.056
110.232
110.122
108.223
PPM
160
150
140
130
120
110
100
90
80
70
60
50
40
30
20
10
0

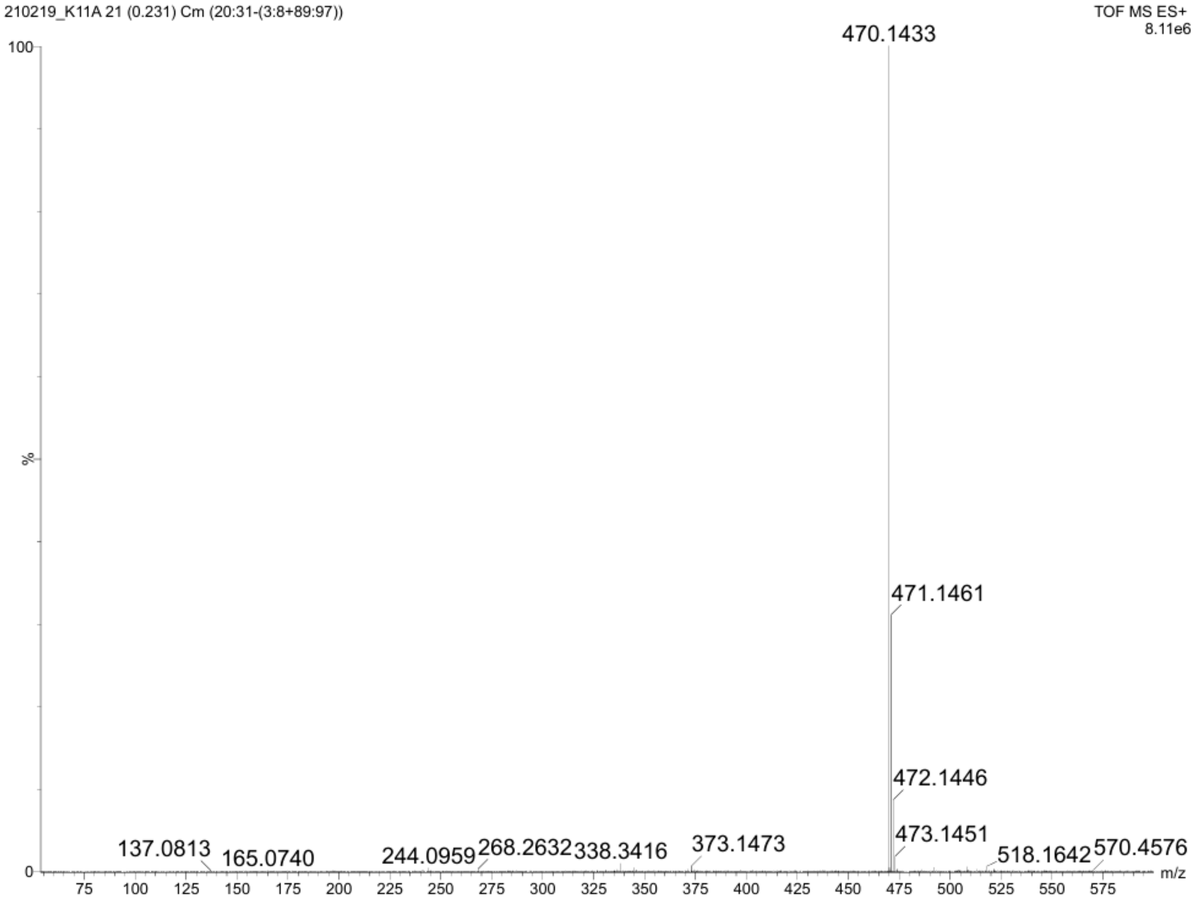
210219_K11A 21 (0.231) Cm (20:31-(3:8+89:97))
TOF MS ES+
8.11e6
100
%
0
470.1433
471.1461
472.1446
473.1451
137.0813
165.0740
244.0959
268.2632
338.3416
373.1473
518.1642
570.4576
m/z

Compound K3

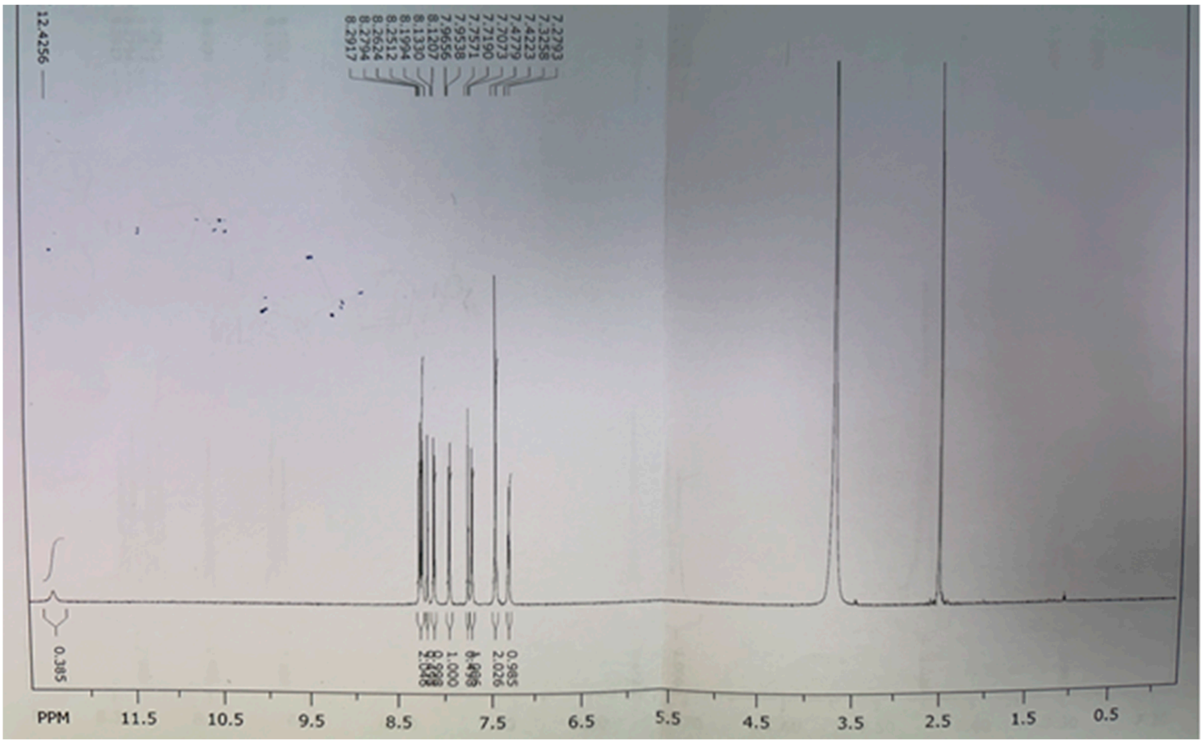
PPM
11.5
10.5
9.5
8.5
7.5
6.5
5.5
4.5
3.5
2.5
1.5
0.5

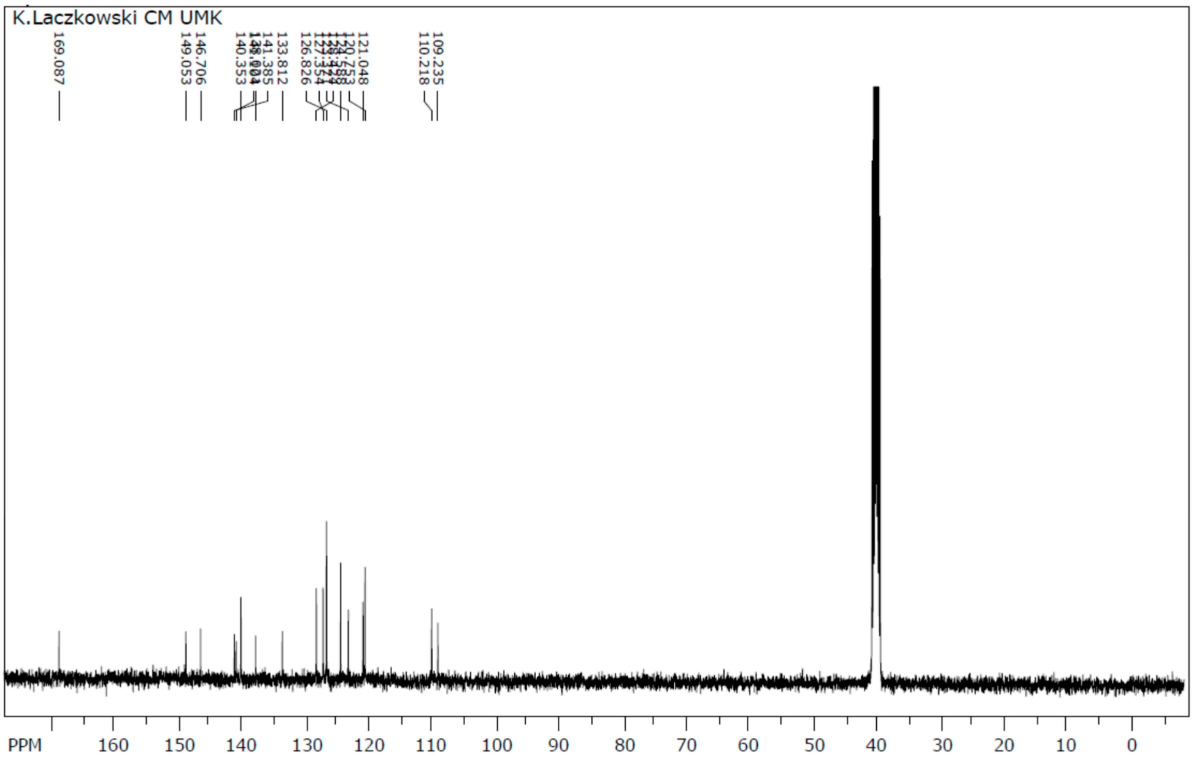
K.Laczkowski CM UMK
169.087
149.053
146.706
140.353
141.385
133.812
126.826
121.048
110.218
109.235
PPM
160
150
140
130
120
110
100
90
80
70
60
50
40
30
20
10
0

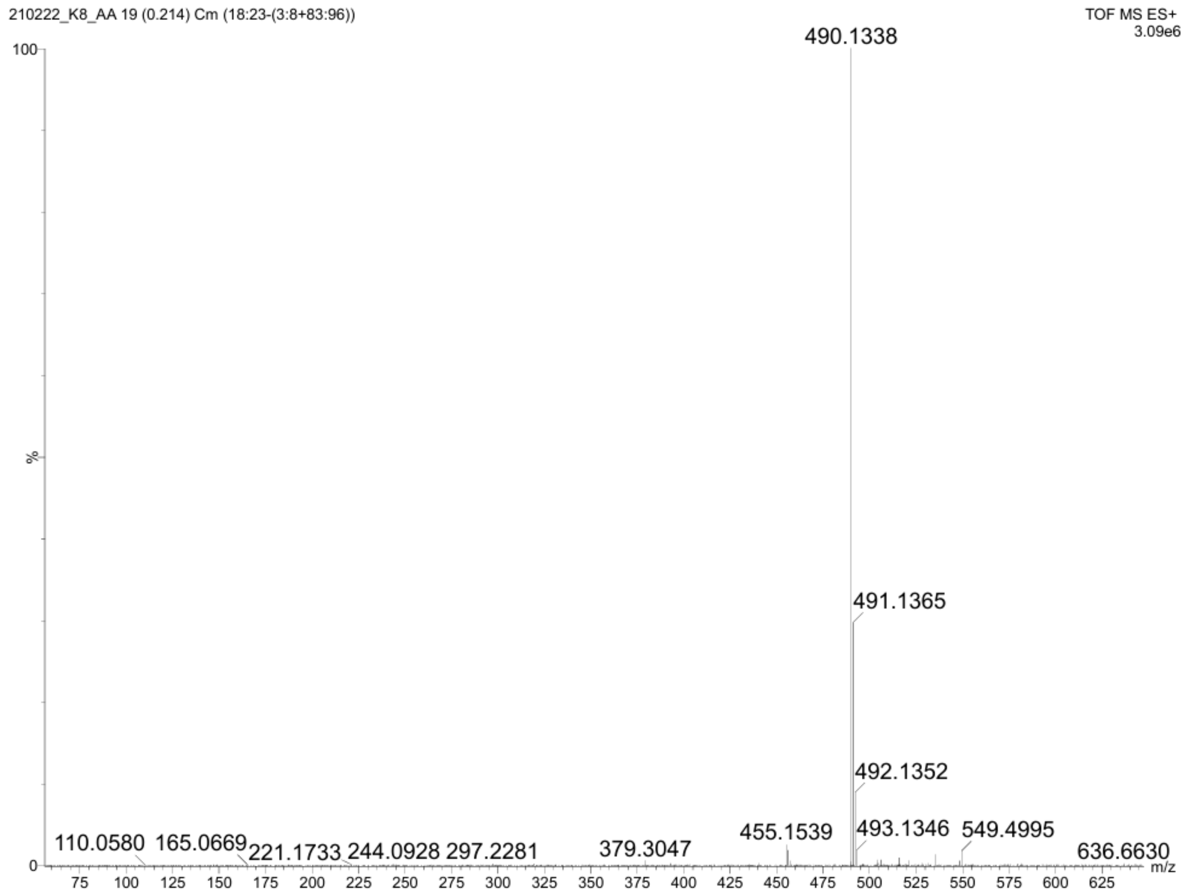

210222_K8_AA 19 (0.214) Cm (18:23-(3:8+83:96))
TOF MS ES+
3.09e6
100
%
0
490.1338
491.1365
492.1352
493.1346
549.4995
636.6630
455.1539
379.3047
297.2281
244.0928
221.1733
165.0669
110.0580
75 100 125 150 175 200 225 250 275 300 325 350 375 400 425 450 475 500 525 550 575 600 625
m/z
